# Discrepancies in classification and reporting of restrictive practices (restraints, seclusion and other coercive measures) in mental health services: multi-scenario analysis of an international survey

**DOI:** 10.1192/bjo.2026.11040

**Published:** 2026-05-11

**Authors:** Zelalem Belayneh, Den-Ching A. Lee, Melissa Petrakis, Deborah Aluh, Justus Uchenna Onu, Giles Newton-Howes, Kim Masters, Yoav Kohn, Jacqueline Sin, Marie-Hélène Goulet, Tonje Lossius Husum, Eleni Jelastopulu, Maria Bakola, Sau Fong Leung, Kathleen De Cuyper, Eimear Muir-Cochrane, Yana Canteloupe, Emer Diviney, Lesley Barr, Jim Ridley, Didier Demassosso, Terry P. Haines

**Affiliations:** School of Primary and Allied Health Care, Faculty of Medicine, Nursing and Health Sciences, Monash University, Melbourne, Australia; Department of Psychiatry, Dilla University, Dilla, Ethiopia; Rehabilitation, Ageing and Independent Living Research Centre, Monash University, Melbourne, Australia; National Centre for Healthy Ageing, Peninsula Health and Monash University, Melbourne, Australia; Mental Health Service, St Vincent’s Hospital, Melbourne, Australia; Lisbon Institute of Global Mental Health, Comprehensive Health Research Centre (CHRC), NOVA Medical School, NOVA University of Lisbon, Lisbon, Portugal; Department of Clinical Pharmacy, University of Nigeria Nsukka, Nsukka, Nigeria; Department of Mental Health, Nnamdi Azikiwe University, Awka, Nigeria; Department of Training and Research, Federal Neuropsychiatric Hospital, Enugu, Nigeria; School of Philological Medicine, University of Otago, Wellington, New Zealand; College of Health Professions, Department of Clinical Sciences, Division of Physician Assistant Studies, Medical University of South Carolina, Charleston, South Carolina, USA; School of Medicine, Jerusalem Mental Health Centre, Hebrew University-Hadassah, Jerusalem, Israel; School of Health and Medical Sciences, City St George’s, University of London, London, UK; Faculty of Nursing, University of Montreal, Montreal, Canada; Research Centre of the University Institute of Mental Health of Montreal, Montreal, Canada; Faculty of Health Sciences, Oslo Metropolitan University, Oslo, Norway; Department of Public Health, Epidemiology and Quality of Life, School of Medicine, University of Patras, Patras, Greece; School of Nursing, The Hong Kong Polytechnic University, Hong Kong, China; LUCAS – Centre for Care Research and Consultancy, University of Leuven, Leuven, Belgium; College of Nursing and Health Sciences, Flinders University, Adelaide, Australia; Eastern Health, Mental Health and Wellbeing, Lived Experience Workforce, Melbourne, Australia; Self Help Addiction Resource Centre, Melbourne, Australia; Curtin School of Nursing, Faculty of Health Sciences, Curtin University, Perth, Australia; Nursing and Governance, Greater Manchester Mental Health Trust, Prestwich, UK; Green Ribbon Health and Community Development Association (GriCoDa), Yaounde, Cameroon

**Keywords:** Restraint, seclusion, restrictive practice, mental health, definition

## Abstract

**Background:**

Global initiatives to reduce restrictive practices in mental health settings have gained increasing attention. However, discrepancies in restrictive practice rates create uncertainties about whether these variations reflect true differences in clinical practices or arise from inconsistent classification and reporting methods.

**Aims:**

This study investigated how healthcare professionals classify and report potential restrictive practice scenarios, and examined variations in classification and documentation across diverse facilities.

**Method:**

This was an international survey conducted using an online questionnaire via the Qualtrics platform. Healthcare professionals working in adult mental health in-patient settings were recruited through multiple media platforms and snowball sampling. The questionnaire included 44 potential restrictive practice case scenarios. Participants rated each scenario as follows: (a) whether it should be classified as a restrictive practice; (b) whether it should be recorded as such; (c) whether it would be classified as a restrictive practice within their facility; and (d) whether it would be reported as a restrictive practice in their facility. Survey development was guided by systematic reviews and co-design work with stakeholders. Data were analysed using ordered regression models, with clustering by participant identity and country. Robust standard errors were applied to ensure accurate estimation of variability.

**Results:**

A total of 491 healthcare professionals from 41 countries participated. Results indicated substantial inconsistencies in clinicians’ perspectives regarding what constitutes restrictive practices and whether a given action should be reported as a restrictive practice. Although participants frequently identified scenarios as restrictive practices, their intention to report them was considerably lower. Additional discrepancies were observed between clinicians’ individual perspectives and their expectations of how these practices were actually being classified and reported as restrictive practices within the in-patient facilities where they work.

**Conclusions:**

Discrepancies between healthcare professionals’ classification of restrictive practices and their reporting intentions, as well as between their perspectives and actual institutional practices, highlight potential errors in current reporting systems. These findings underscore the need for standardised definitions, enhanced reporting frameworks and structured training programmes and monitoring mechanisms to improve consistency in the management of restrictive practices across mental health settings.

Restrictive practices are interventions, such as physical/mechanical restraints, chemical restraints, seclusion, involuntary treatment and other coercive measures that limit an individual’s autonomy or choice, freedom of movement and ability to act independently.^1^ These practices are predominantly used in mental health settings, and their use is often justified as a safety measure to manage acute crises when individuals’ behaviours are perceived to pose imminent risks of danger to themselves or others.^2^ The use of restrictive practices has long been a topic of significant ethical, legal and clinical debate due to the physical and psychological harms they can cause.^3^ Restrictive practices are now considered a breach of human rights and safety that must be urgently avoided. The World Health Organization (WHO) has excluded seclusion and restraint from its classification of healthcare interventions, explicitly considering them as non-therapeutic responses .^4^

## Reduction of restrictive practice use

Over the past two decades, there have been growing initiatives to reduce, and eventually eliminate the use of restrictive practices and promote least-restrictive alternatives. The adoption of the United Nations Convention on the Rights of Persons with Disabilities in 2006^5^ marked a pivotal shift, framing the elimination of restrictive practices in mental healthcare services as a human rights obligation. Several countries, including Australia,^6^ the UK,^7^ New Zealand,^8^ Germany^9^ and Canada,^10^ have implemented policy frameworks advocating non-restrictive, recovery-oriented approaches to crisis management.^4^ These frameworks have been supported by evidence-based initiatives such as the Open Wards Policy,^11^ the Six Core Strategies,^12^ the Safewards model^13^ and trauma-informed care, all of which aim to reduce or eliminate restrictive practices.^14^

Despite these initiatives, significant variability in the reported prevalence rates of restrictive practice use continues to impede efforts to accurately monitor and evaluate the effectiveness of reduction strategies and policy reforms.^15^ For example, one study^16^ reported mechanical restraint rates vary from 0.03 to 98.8%. A recent systematic review^17^ also highlighted substantial inconsistency in restrictive practice prevalence rates: physical restraint ranged from 0.3 to 54%, chemical restraint from 1 to 58% and seclusion from 2 to 56%. Such discrepancies adversely limit the empirical utility of existing data for benchmarking, ongoing monitoring and evaluation of restrictive practice use to understand the true extent of restrictive practice use and to track its trend over time.^18^ Emerging evidence suggests that these differences may not be fully explained by actual variations in the frequency of implementation of restrictive practices; instead, these discrepancies may partly stem from inconsistent classification and reporting methods.^19^

## Classification and reporting of restrictive practices

Many jurisdictions now consider the use of restrictive practices as reportable clinical incidents within their legal and institutional governance systems.^19,20^ However, the lack of universal definitions and consistent reporting mechanisms complicates accurate classification and reporting practices.^21^ Healthcare professionals often rely on their personal judgements (which often are further shaped by several cultural and organisational factors) to determine whether a given action constitutes a restrictive practice and whether it requires formal documentation in the hospital reporting system.^16^ The few central repositories that exist typically recognise only physically overt restrictive practices, such as physical restraint, seclusion and mechanical restraint (and in some contexts, chemical restraint), with documentation mandated particularly for these practices.^19^

Some organisations, such as WHO and the World Mental Health Atlas, recognise physical restraint, seclusion and mechanical restraints (sometimes also chemical restraints), whereas consumer organisations (e.g. the Victorian Mental Illness Awareness Council in Australia) identify restrictive practices as any intervention aimed at changing the behaviour of service users.^22^ Similarly, Bennett et al^23^ reported that healthcare practitioners often view restrictive practices as necessary to maintain control of the ward environment, whereas patients frequently described them as actions creating feelings of being controlled and a lack of safety, which constitute violations of their human rights. These inconsistencies in definitions and classification of restrictive practices between organisational policy and service user perspectives remain a source of ongoing debate, hindering collaborative efforts among multiple stakeholders to reduce the use of restrictive practices.^24^

The classification and documentation of restrictive practices in mental health in-patient settings can be influenced by several factors, including hospital policies, flaws in reporting systems, staffing levels and the clarity and comprehensiveness of incident-reporting mechanisms.^25^ Additionally, the severity and symptom presentation of patients, clinicians’ skills, level of training and their prior exposure to restrictive practices play roles in their classification and reporting behaviours. Contextual factors such as the duration of the action, consent status (i.e. whether the restrictive practice was administered against the person’s will), methods applied (human force, device, medication or verbal threats), clinicians’ intentions, legal attributes and timing when the action takes place may further exacerbate these discrepancies.^26^ For example, hospital staff may fail to report restrictive practices that occur during shift changes, or may document them only in medical charts rather than in formal incident-reporting forms, often due to time constraints.^27^ Such documentation errors are likely to introduce biases in the reported data, potentially distorting the actual prevalence of restrictive practice use and misleading decision-making.^19,20^

Despite this fact, most studies assessing and comparing the prevalence of restrictive practices continue to rely on secondary data sources such as patient medical records/notes, routinely collected by healthcare professionals (HCPs),^17^ even though little is known about how professionals classify and record restrictive practices. This undermines the reliability and trustworthiness of these studies in making valid comparisons and drawing meaningful conclusions based on the evidence generated.^28^

The purpose of the current study was to examine how HCPs classify and report potential restrictive practice scenarios in adult mental health in-patient settings worldwide. It also explored the classification and reporting patterns for each scenario, highlighting differences in interpretation and documentation behaviours. Specifically, the study examined differences in HCPs’ responses regarding the following: (a) whether they think each scenario describes a case of a restrictive practice; (b) whether they think each scenario should be documented as a restrictive practice in their facility’s reporting system; (c) whether they think each scenario would actually be recognised and classified as a restrictive practice at their facility; and (d) whether they think that each scenario would actually be documented and reported as a restrictive practice within their facility.

## Method

### Study design and participants

This study employed a cross-sectional design using an online questionnaire administered globally. The survey was conducted via the Qualtrics platform and targeted healthcare professionals who self-identified as working in adult mental health in-patient settings across multiple countries. Adult mental health in-patient services generally provide intensive care and treatment for individuals aged 18–65 years with acute psychiatric conditions requiring close monitoring that cannot be managed in out-patient or community-based settings.

### Inclusion and exclusion criteria

Eligible participants included psychiatrists, medical officers, general nurses, psychiatric nurses, psychologists, social workers, occupational therapists and other trained and qualified clinicians working in adult mental health in-patient facilities. The study excluded professionals who worked only in (a) out-patient departments, (b) forensic settings, (c) geriatric or podiatric in-patient units, (d) non-mental health contexts (e.g. neurological or developmental disorders) or (e) home care settings, unless they also had additional experiences of working in adult mental health in-patient settings. This exclusion criterion was applied to ensure that all participants responded to case scenarios within a shared clinical context, thereby maximising the comparability of responses and avoiding conflation of sector-specific differences in practices, legal definitions and reporting requirements.^3^

### Survey development

The survey was co-designed through a collaborative process involving 33 team members from 17 countries across Africa, Asia, North America, Europe and Australia. The team included various mental health stakeholders, including clinicians, researchers, patients, family caregivers and mental health educators. Drawing on insights from our prior systematic reviews,^17,29,30^ the team proposed the use of case scenarios as a method to collect survey data. Across 6 panel discussions, the team identified 23 distinct contextual categories that reflect real-world experiences of how restrictive practices are applied in adult mental health in-patient facilities (Supplementary File 1). These scenarios are considered factors affecting clinicians’ decission-making in determining whether or not a given action constitutes a restrictive practice, and whether it should be documented as such in the hospital reporting system. Based on these contexts, the co-design team members co-developed 81 specific case scenarios illustrating the various situations in which restrictive practices commonly manifest in adult mental health in-patient services. Full methodological details of the co-design process are reported elsewhere.^31^

In this study, restrictive practices are broadly defined to include any action or intervention (explicit or implicit) that limits a person’s rights, freedom of movement, choice or ability to act independently, and includes non-visible informal coercions. The case scenarios we developed involved mechanical restraint (*n* = 21), seclusion (*n* = 16), chemical restraint (*n* = 12), environmental restriction (*n* = 7), manual holding (*n* = 6), psychological/emotional restraint (*n* = 4), involuntary intervention (*n* = 4) and combined methods (*n* = 11).

Within each context, multiple parallel and comparable scenarios that share identical core contexts but varied only in specific situational elements were developed. This approach has been chosen to assess in isolation the influence of individual contextual elements on participants’ classification and/or reporting intentions of a given action as a restrictive practice.

For example, in Context 7 (trial of less restrictive alternatives), three parallel scenarios were constructed as follows:

- The medical team applies mechanical restraint to a person displaying aggressive behaviours during hospital admission after all available less restrictive alternatives had been attempted and found ineffective.
- The medical team applies mechanical restraint to a person displaying aggressive behaviours during hospital admission after some less restrictive alternatives were attempted, resulting in partial or insufficient clinical benefit.
- The medical team applies mechanical restraint to a person displaying aggressive behaviours during hospital admission without attempting other less restrictive alternatives, based on the clinician’s belief that restraint was the only available approach to achieve the desired outcome.

In these scenarios, only the statements describing the extent to which less restrictive alternatives were attempted varied within the context, whereas all other scenario elements were held constant across the three descriptions. The development of all other scenarios within each context followed a similar approach (Supplementary File 1).

Drafted scenarios were refined through multiple iterative expert review, and consensus was reached among the panel members. The co-design team members then ranked the 81 scenarios based on their relevance and likelihood of occurrence in real-world clinical settings across various regions and prioritised a final set of 44 scenarios to be included in the survey data collection. Full descriptions of these scenarios are provided in Table 1. To support international recruitment and participation, the survey was professionally translated into Amharic, Hebrew, Greek, French and Dutch languages. Each translation was validated and pre-tested by research team members from the respective regions, ensuring linguistic and contextual accuracy of the survey.

**Table 1:**
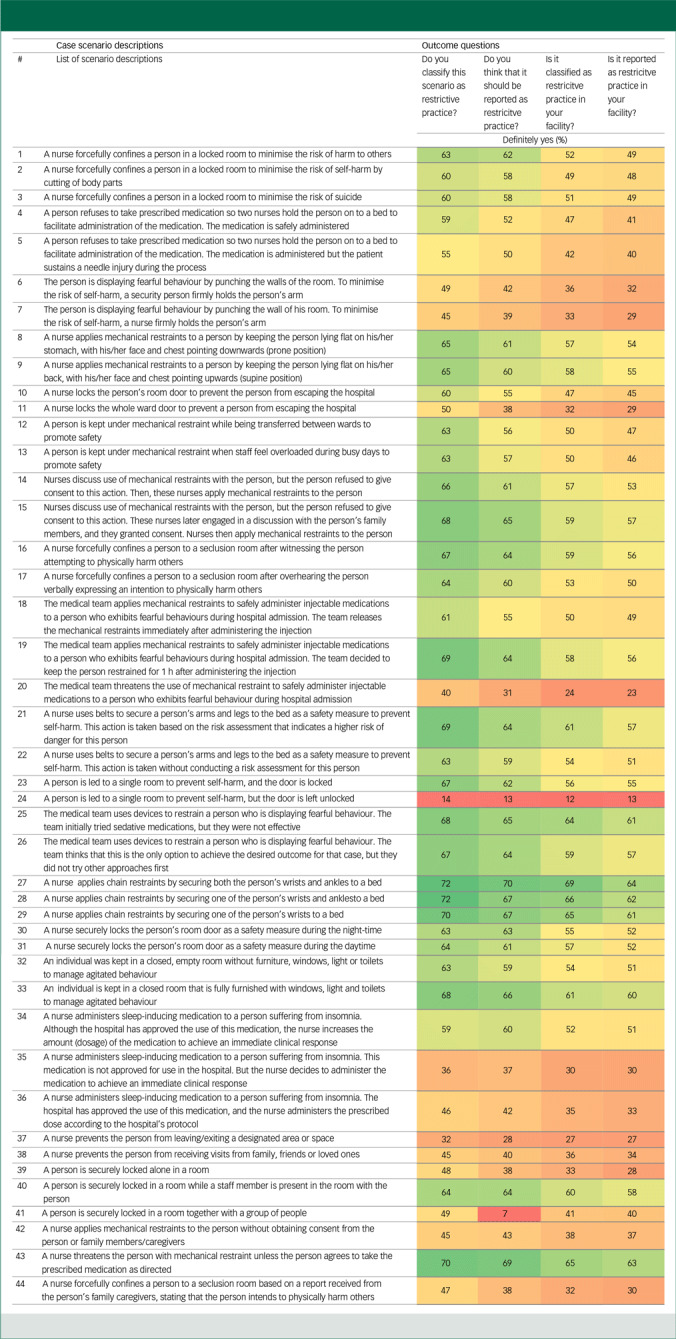
A declining pattern in the proportion of healthcare professionals’ ‘definitely yes’ responses for the classification and reporting of scenarios as restrictive practices across the four outcome questions

RP, restrictive practices; S. no, scenario numbers.

Change in cell colour from dark green to red indicates declining patterns in participants’ responses across the four outcome questions.

### Recruitment procedure

Participants were recruited using a non-probability sampling approach. The survey questionnaire was hosted online via the Qualtrics platform^32^ and disseminated widely by sending invitation through organisational mailing lists, releasing in e-newsletters and by posting it on institutional websites. Dissemination was supported by several professional and research networks, including the LUCAS–Centre for Care Research & Consultancy (Belgium), Integrated Mental Health for Health Professionals (Australia), the Ethiopian Mental Health Professionals’ Association (Ethiopia), FOSTREN (Europe) and Te Pou and Blueprint for Learning (New Zealand). Snowball sampling was also employed by encouraging participants to share the survey within their professional networks.

Members of the international research team further promoted both the English- and local-language versions of the survey through social media platforms (LinkedIn, Facebook and X), and served as regional contact persons to support data collection within their respective regions.

Participant recruitment continued from November 2024 to February 2025 (the entire period during which the survey was available online). In total, 673 healthcare professionals accessed the survey. Following data-cleaning procedures, which excluded incomplete data, responses from 491 participants were included in the final analysis and presented in this paper.

### Outcome measurement

The primary outcome of this study (classification and reporting of potential restrictive practice scenarios) was measured using participants’ ratings of each scenario across the following four questions:

- whether the participant believed the actions described in the scenario constitute a restrictive practice;
- whether the participant believed it should be reported as a restrictive practice in the hospital’s reporting system;
- whether the scenario was actually being classified as a restrictive practice within their facility;
- whether it was actually being documented as a restrictive practice within the facility’s reporting system.

These outcome questions were designed to assess and compare discrepancies between clinicians’ perceived classification of scenarios and their reporting intentions, as well as variations between their personal views on what should be reported and their expectations of actual hospital reporting practices. The first question focused on clinicians’ personal perspectives and their level of recognition and identification of each scenario whether or not as a restrictive practice. The second question examined whether participants believed the action described should be documented as restrictive practice in the hospital’s reporting system. This question was designed to determine whether, and to what extent, clinicians intend to report actions they already recognise as restrictive practices, thereby identifying potential discrepancies between recognition and reporting intentions. The third question explored participants’ expectations and experiences in their workplaces regarding whether the action described in each scenario is legally recognised and defined as a restrictive practice. The fourth question examined whether the scenario was actually being documented and reported as a restrictive practice within the reporting systems of their hospital or workplace settings. This provided insight into the proportion of scenarios identified as restrictive practices that would probably be formally reported, and offered indicative evidence of how organisational and systemic factors may influence clinicians’ reporting behaviour, even when they recognise actions as reportable restrictive practice incidents.

For each outcome question, participants responded using a four-point Likert scale (1 = definitely yes, 2 = probably yes, 3 = probably no and 4 = definitely no) response option (Supplementary File 2). This approach not only enabled the assessment of variations in how restrictive practices are classified and reported across different mental health facilities, but also captured the degree of uncertainty and confidence in participants’ responses, reflecting the challenges clinicians often face when classifying and reporting restricitve practice actions.

### Ethical standards

The authors affirm that all procedures contributing to this work comply with the ethical standards of the relevant national and institutional committees on human experimentation, and with the Helsinki Declaration of 1975, as revised in 2013. This study has been ethical reviewed and approved by Monash University Huma research Ethics Committee (Project ID: 3642). Informed consent was obtained from all participants through an online consent statement presented prior to accessing the survey. Only participants who provided consent were permitted to proceed to the survey questions; the survey was automatically terminated for those who did not consent. Participants were clearly informed in the consent statement that their responses would be de-identified and reported in an anonymised form by the authors’ publications and other dessemination proceduers.

### Analysis

Descriptive statistics, including frequencies and percentages were calculated to summarise participant characteristics and to compare the proportions of HCPs who classified and reported each case scenario as restrictive practice. Separate analyses were conducted for each scenario across the each of the four outcome questions: perceived classification, personal reporting intentions, expectation of actual institutional classification and expectation of actual institutional reporting practices.

Participants’ responses across the four outcome questions were compared using ordered logistic regression models:

- Comparison 1: participant personal perception of what should be classified as restrictive practice (Question 1) versus what should be reported (Question 2);
- Comparison 2: participant expectations of what was being classified as restrictive practice within their facility (Question 3) versus what was actually being reported as such in their facilities (Question 4);
- Comparison 3: participant personal perception of what should be classified as restrictive practice (Question 1) versus what was actually being classified as such within their facilities (Question 3);
- Comparison 4: participant personal perception of what should be reported as restrictive practice (Question 2) versus their expectations of what was actually being reported within their facility (Question 4);
- Comparison 5: participant personal perception of what should be classified as restrictive practice (Question 1) versus thier expectation of what was actually being reported within their facility (Question 4).

These models estimated the odds of providing higher versus lower ratings on the four-point ordinal scale (‘Definitely yes’, ‘Probably yes’, ‘Probably no’, ‘Definitely no’). Odds ratios with 95% confidence intervals were reported to facilitate interpretation of differences in clinicians’ responses across paired comparisons of their response for the four outcome questions. All analyses were performed using STATA version 17.

Robust standard errors, clustered at the participant level were applied to account for repeated responses across multiple scenarios,^33^ and data were further clustered by countries and regions.^34^ Any missing responses were addressed using complete-case analysis and multiple imputation to minimise bias in the estimates. The proportional odds assumption for all models was tested using the Brant test and the assumption was met for each model.

## Results

### Characteristics of study participants

Responses from 491 HCPs from across 5 regions: Africa (39.26%), Europe (34.18%), Asia (8.59%), North America (3.52%) and Australasia (14.71%) reported in this paper. In terms of professional roles, nurses represented the largest group (46.47%), followed by medical doctors (22.73%), psychologists (12.63%), medical officers (5.05%), social workers (3.03%), occupational therapists (1.52%) and other trained and qualified professionals (8.59%). Demographically, 38.90% of participants were between 31 and 40 years old, and 36.36% had over 10 years of work experience in mental health.

### Frequency of scenario occurrence

From a total of 491 participants who fully completed the 22 core-item scenarios, 197 (40.25%) also responded to the additional optional-item questions. Healthcare professionals from all regions reported the occurrence of every scenario in their workplace at different frequencies. More than half of participants indicated that 31 out of the 44 scenarios (70%) occurred at least once a year. Among these, scenarios involving the forceful confinement of a person in a locked room, holding a person on a bed, firmly holding a person’s arms, isolating a person after they attempted to physically harm others, administering medication for sedation purposes and preventing a person from leaving a designated area, were particularly mentioned as more frequently occuring practices. Over 75% of HCPs reported witnessing these actions at least once a year, and at least 50% observed them once a month or more frequently (Supplementary File 3).

### Variations in classifying and reporting of restrictive practices

A summary of the raw data for participants’ responses across each of the four questions where they were asked to rate in terms of their level of agreement on the classification and reporting of scenarios whether or not as restrictive practices is presented in Supplementary File 4. The analysis revealed inconsistencies in how HCPs across mental health in-patient settings classified and documented various case scenarios as restrictive practices, as well as how these practices were actually being classified and reported in their facilities. The data show a declining trend in healthcare professionals’ responses; many clinicians responded ‘definitely yes’ for their perceived classification (Q1), while the proportion shows a systematically declining pattern when we move to personal reporting intentions (Q2), actual institutional classification (Q3) and finally actual institutional reporting (Q4) for each scenario.

Findings from the five comparisons across the four outcome questions are summarised as follows:

#### Comparison 1: clinicians’ perceived classification (Question 1) versus perceived reporting intentions (Question 2)

**Fig. 1:**
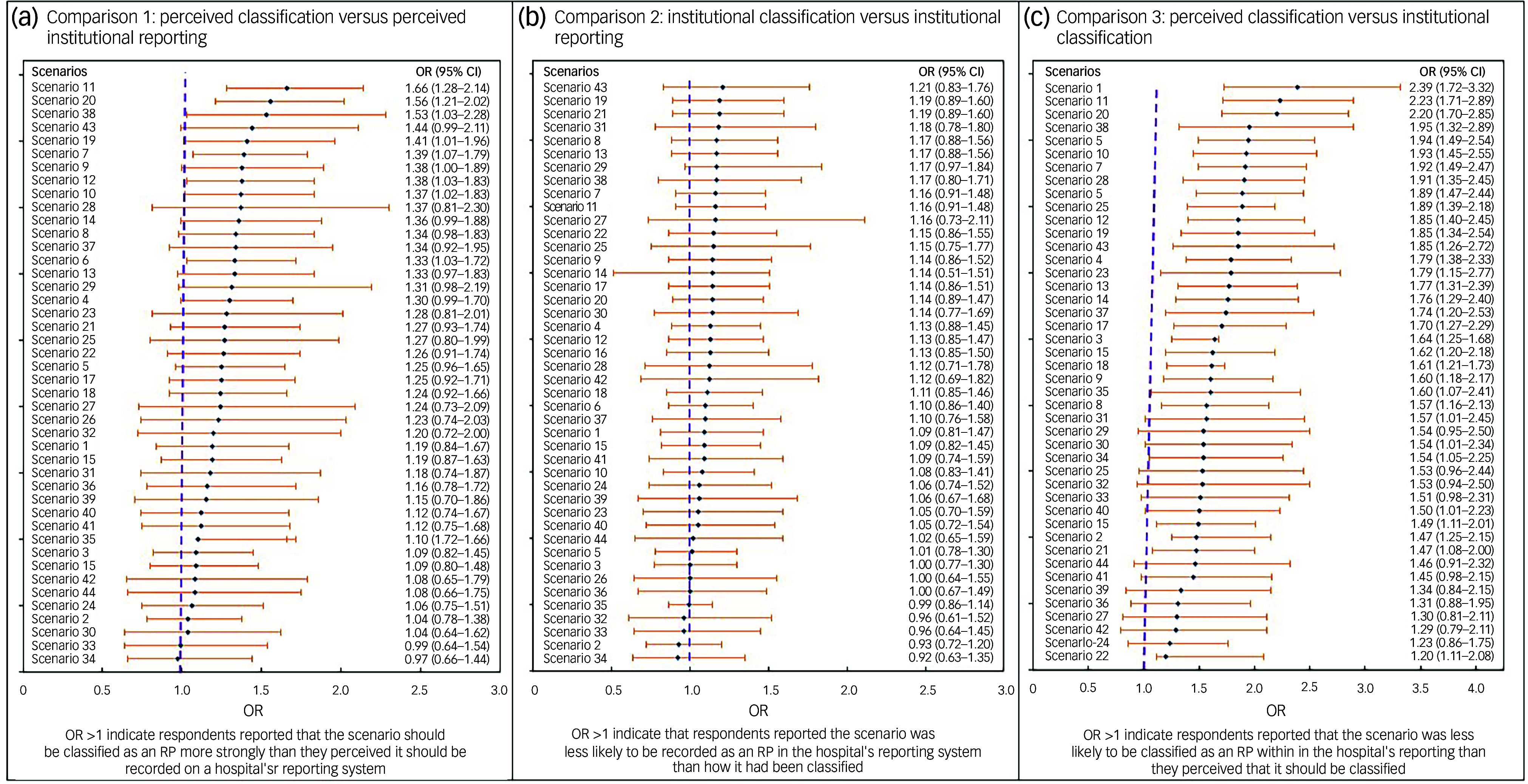
Forest plots comparing participants’ responses across different outcome measures: (a) what should be classified as RP versus what should be reported as RP; (b) what is actually being classified as RP versus what is actually being reported as RP; (c) what should be classified as RP versus what is actually being classified as RP. OR, odds ratio; RP, restrictive practice.

Healthcare professionals generally expressed a greater intention to recognise and classify more scenarios as restrictive practices than to record and report them in the hospital system (Table 1). For example, in a scenario involving locking the whole ward door to prevent a person from escaping the hospital while leaving individual room doors open (Scenario 11), 220 respondents (50%) classified this scenario as a restrictive practice; however, only 166 (38%) indicated that they would document it as a restrictive practice in the hospital incident-reporting system. These discrepancies resulted in an odds ratio of 1.66 (95% CI: 1.28–2.14), indicating a substantial decline in the likelihood of documentation and reporting of restrictive practices despite professionals’ recognition of the practice as restrictive. Statistically significant differences between Questions 1 and 2 were observed in 10 out of 44 scenarios (22.72%) (Fig. 1(a)).

#### Comparison 2: institutional classification (Question 3) versus institutional documentation (Question 4)

This comparison indicated that not all scenarios considered restrictive practices by the hospital protocols and guidelines would actually being documented as such in the hospital reporting (Fig. 1(b)). In a scenario involving locking the whole ward door to prevent a person from escaping the hospital while leaving individual room doors open (Scenario 11), 140 participants (31.96%) indicated that their facility would classify the action as a restrictive practice. However, only a smaller proportion (28.5%) indicated that it would actually be documented (Table 1). Conversely, 100 respondents definitely disagreed with the idea of formal documentation, compared with 89 who disagreed with the classification.

#### Comparison 3: clinicians’ perceived classification (Question 1) versus institutional classification (Question 3)

Inconsistencies were observed between participants’ perspectives on what should be classified as a restrictive practice and their views on whether the same scenario was actually being classified as such in their facilities. Across all scenarios, a higher proportion of clinicians classified scenarios as restrictive practices than those who believed their facility would actually classify them as restrictive practices. For example, in a scenario involving locking the whole ward door to prevent a person from escaping the hospital while leaving individual room doors open (Scenario 11), 220 participants (50.2%) definitely agreed that the scenario should be classified as an action but only 140 (31.96%) felt that their facilities would actually classify it in the same way within their workplace (Table 1). This classification difference yielded an odds ratio of 2.23 (95% CI: 1.71–2.89). Statistically significant differences were observed in all 44 scenarios compared (Fig. 1(c)).

#### Comparison 4: clinicians’ perceived reporting intentions (Question 2) versus actual institutional documentation (Question 4)

**Fig. 2:**
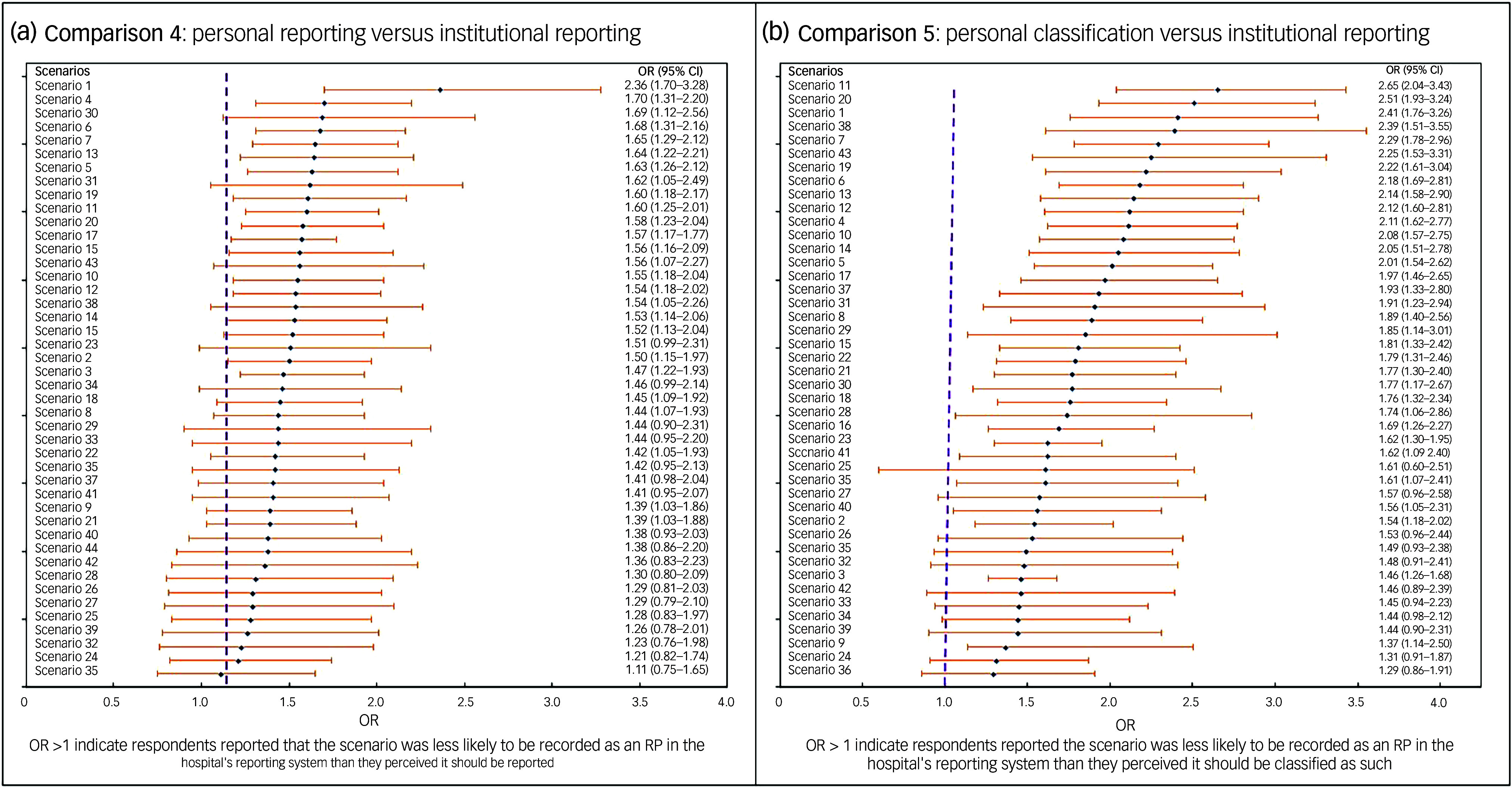
Forest plots comparing participants’ responses to the two outcome measures: (a) what should be reported as RPs versus what is actually being reported as RP; (b) What should be classified as RP versus what would actually being reported as RPs. OR, odds ratio; RP, restrictive practice.

This comparison examined inconsistencies between HCPs’ intentions of reporting scenarios as restricitve practices and their expectation of actual hospital reporting practices. The data showed that, whereas they often agreed that many of the scenarios should be reported as restrictive practices, their level of agreement was significantly lower when asked whether these scenarios would actually be reported as such in their facilities. For a scenario involving locking the whole ward door to prevent a person from escaping the hospital while leaving individual room doors open (Scenario 11), 166 participants definitely agreed that the scenario required formal documentation as a restrictive practice (Table 1). However, only a smaller proportion (*n* = 125) indicated that it would definitely be reported as a restrictive practice in their in-patient facility, resulting in an odds ratio of 1.60 (95% CI: 1.25–2.01). This indicates that, even if clinicians recognise the need for documentation of restrictive practice episodes, institutional or systemic barriers may inhibit actual reporting practices. All scenarios demonstrated statistically significant differences in clinicians’ personal reporting intentions and their expectation of actual reporting pratices and the same restrictive practices in their facilities (Fig. 2(a)).

#### Comparison 5: clinicians’ perceived classification (Question 1) versus actual institutional documentation (Question 4)

The final comparison highlighted the most profound discrepancy between HCPs’ personal classification and recognition of scenarios as restrictive practices and their expectations about whether the hospital system would actually recognise and document these scenarios. For example, in a scenario involving locking the whole ward door to prevent a person from escaping the hospital while leaving individual room doors open (Scenario 11), 220 (44.8%) participants definitely classified this scenario as restrictive practice. Their response regarding whether or not this action would actually be documented as such within their facility’s reporting system decreased to 125 (24.5%) (Table 1). On the contrary, 100 participants stated that they would definitely not consider the scenario restrictive practice, compared with just 57 participants who definitely disagreed with its classification as a restrictive practice. This disparity generated the highest odds ratio of 2.65 (95% CI: 2.04–3.43) (Fig. 2(b)).

#### Result summary

**Fig. 3:**
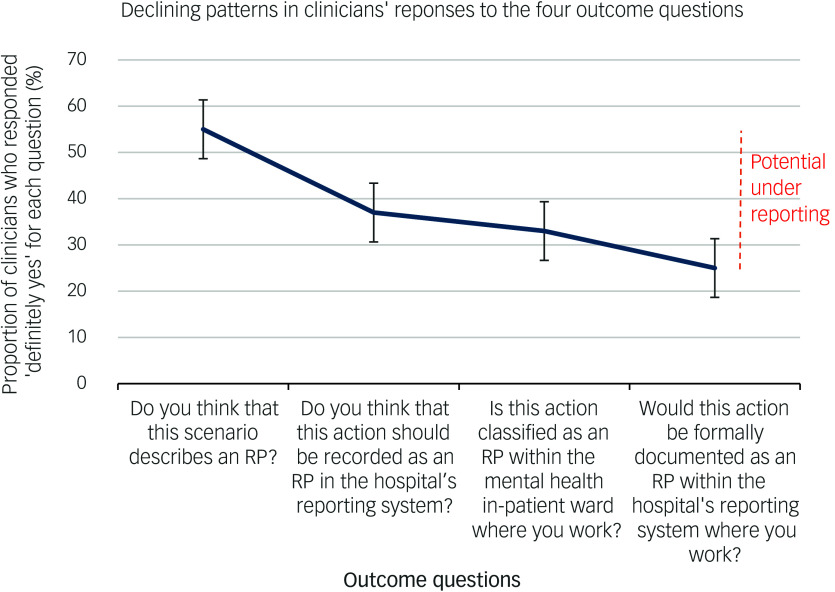
A sample diagram illustrating a systeamtically declining patterns in participants’ responses from their personal recognition of scenarios as restrictive practices to their expectations of actual hospital reporting practices as RP. RP, restrictive practice.

The overall findings identified a systematic declining pattern in clinicians’ responses to the four outcome questions, from Question 1 to Question 4, indicating a potential for underreporting (Fig. 3). Using Scenario 11 (the example scenarios illustrated above), 220 classified it as restrictive practice (Q1), 140 said that it should be reported (Q2) and 140 believed that their facility would report it (Q3). Furthermore, 125 indicated that this action would actually be documented as restrictive practice in their facility’s reporting system (Table 1).

## Discussion

The current research provides compelling empirical evidence of systematic underreporting of restrictive practices, demonstrated by substantial discrepancies in how HCPs classify and report restrictive practices. The findings indicate inconsistencies between clinicians’ personal judgements about the classification and reporting of restrictive practices and their expectations of the actual practices within the in-patient mental health facilities where they work. An overriding pattern emerges in clinicians’ response, suggesting that the scenarios presented were more commonly recognised as restrictive practices (Question 1), yet fewer HCPs believed that these scenarios should be reported (Question 2). Even fewer thought that these practices would actually being recognised and classified as restrictive practices within their facility (Question 3), with the least agreement observed regarding whether these actions were actually being documented as restrictive practices within their reporting systems (Question 4). Collectively, these findings implicitly suggest that HCPs perceive systemic underreporting of restrictive practices in mental health in-patient facilities.

One previous systematic review found significant variability in reported rates of restrictive practices, with this variability having been further complicated by differences in how restrictive practices are defined and measured.^17,19,30^ Our research adds empirical evidence, that this is highly likely to be the case. Many participants in the current study appeared to be hesitant to document scenarios as restrictive practices, even when they personally considered them to be restrictive, largely due to unclear institutional reporting requirements and lack of comprehensive reporting mechanisms. Participants frequently reported uncertainty about whether such practices would actually being classified and reported as restrictive practices within their facilities, suggesting a potential gap between individual professional judgement and institutional reporting practices.^18,35^ The findings highlight how external factors, such as hospital policies, institutional norms, fear of professional repercussions, administrative hurdles and work culture can substantially influence clinicians’ documentation and reporting behaviours.^36^

Importantly, legal definitions of restrictive practices in many jurisdictions tend to focus primarily to physically visible practices, implicitly leaving clinicians with no or little obligation to document less visible, yet equally harmful, coercive practices.^37^ Such legal and policy oversights may contribute to a systemic underreporting and create ambiguity in understanding the true prevalence of restrictive practice use, as well to as the monitoring and evaluations of effectiveness of policies and interventions aimed at reducing these practices.^38^ Contextual factors, including locked versus unlocked doors; actual implementation of actions versus only threatening the person with the action; differences in healthcare professional intentions; duration of the action (short versus long); and the type of person applying the action (e.g. nurse versus security staff) showed inconsistencies in participants’ recognition of restrictive practices actions and their intentions to report intentions. Establishing consistent definitions, comprehensive classification taxonomies and reliable reporting frameworks that integrate the full spectrum of restrictive practices, including hidden and less visible forms that are hard to detect and regulate. Such approaches support consistency of care, often applying principles of continuous quality improvement in healthcare. Ultimately, such frameworks support more accurate monitoring of restrictive practices, facilitate the identification of risks and promote safer, more ethical and patient-centred mental health care.

Savage et al^19^ revealed that the types and numbers of reported restrictive practices reported across national databases from nine countries varied widely, and that the measurement indicators applied across each data-set were inconsistent. These inconsistencies compromise both the quality of clinical care and the integration of restrictive practice reduction strategies into routine clinical practice.^18^ A useful first step towards achieving consistent documentation and reliable data reporting would be to employ a standardised definition and classification framework.^18^ However, achieving consensus is a long-term plan, and research in the related field has shown that simply providing common definitions to hospital staff does not fully improve consistency in incident classification and reporting practices.^27^ Thus, interim complementary approaches are needed, such as structured training alternatives on how to apply common definitions in various contexts, as well as the establishment of transparent institutional policies, monitoring systems and evaluation strategies.^26^ Integrating restrictive practice coding systems into existing global frameworks, such as the WHO Mental Health Atlas and the International Classification of Diseases could further enhance consistency.

### Implications

This research provides evidence for the potential underreporting of restrictive practice incidents. The extent of this underreporting can be assessed by examining scenarios with high levels of uncertainty about whether they constitute a restrictive practice, in conjunction with additional data on how frequently each scenario was observed in in-patient facilities. For example, 72% of our sample reported that Scenario 4 (involving the forceful administration of a prescribed medication) occurred in their facilities at least once a month. However, 21% of HCPs indicated that this scenario would probably not be recorded as a restrictive practice, and 12% stated that it would definitely not be recorded in their hospital’s reporting system, demonstrating a clear gap between the frequency with which such practices occur and the likelihood that they are formally documented as restrictive practices. This discrepancy between reported practices and those experienced in clinical settings also suggests potential differences between what is formally recorded and what service users may actually experience in in-patient environments. As a result, vulnerable populations may remain at continued risk of harm despite growing international initiatives aimed at minimising and eliminating restrictive practices.

Overall, the findings highlight the need for agreed-upon updates to definitions, standardised classification taxonomies and consistent, reliable documentation and reporting frameworks for restrictive practices. Such frameworks could include the integration of stand-alone considerations of restrictive practices into existing classification and reporting systems – for example, within occupational health, national ICD-10 coding or mental health information systems. This would allow clinicians across different regions to objectively identify and consistently report actions, whether or not these are currently recognised as restrictive practices. Although full standardisation may be a long-term goal, interim measures such as targeted training, alternative reporting approaches and monitoring systems could be implemented concurrently to support more consistent classification and documentation in the short term.

This study did not directly measure the actual rates of underreporting due to the cross-sectional nature of the study. A formal evaluation of this would require the application of standardised definitions and a concurrent examination of multiple sources of truth. Methods, such as medical record reviews, direct observations, staff self-reports, incident reporting systems and feedback from people who use the service and caregivers could help in accurately quantifying underreporting.^27^ However, HCPs may change their patterns of restrictive practice use, classification decisions and documentation behaviour if they know that they are being observed, potentially yielding biased data. Controlled trials or the use of video recording would more accurately capture underreported incidents,^39^ but these approaches necessitate careful consideration of ethical and safety concerns due to the sensitive nature of mental health in general and restrictive practices in particular.

### Limitations

The use of restrictive practices appears to occur across many sectors supporting vulnerable populations, including forensic care, aged care, disability services, school environments, community services, legal systems and other healthcare settings. These sectors and jurisdictions may apply differing policies and varying legal definitions for applying, classifying and reporting restrictive practices, limiting the generalisability of the findings of this research beyond adult mental health in-patient settings. For instance, data collected from clinicians working in locked or forensic wards may differ substantially from those of individuals working in general mental health wards. Expanding similar research to include these diverse sectors would enable valid cross-sector comparisons and promote shared learning on best practices for effective reduction of restrictive practices.^40^ Such an approach would facilitate the development of more consistent, cross-context methodologies and multisectoral collaborations, supporting the transition toward more recovery-oriented and least-restrictive, evidence-based approaches to care.

The use of snowball and other non-probability sampling approaches may have introduced selection bias, as participant recruitment relied on professional networks and voluntary engagement with the survey. Consequently, individuals with greater interest or awareness of restrictive practices are more likely to participate, potentially influencing the distribution of responses. In addition, the number of participants varied across countries and professional groups, which may have limited the representativeness of the sample and limit balanced cross-country comparisons using data generated. Differences observed between jurisdictions should therefore, be interpreted with caution, because these may reflect contextual variations in policies and reporting systems, as well as uneven sample representation. Accordingly, the findings should not be interpreted as definitive estimates of restrictive practice classification or reporting practices across all mental health in-patient settings, but rather as indicative insights into clinicians’ perspectives and perceived discrepancies between recognition and reporting of restrictive practices. Moreover, the multiple scenario comparisons conducted in the analysis may increase the risk of false-positive results. To account for the data structure and heteroscedasticity, clustering by participant ID and country, along with robust standard errors, was applied in the analysis. However, formal adjustment for multiple comparisons was not performed due to the explorative nature of the study, consistent with best practices for hypothesis-generating. Nonetheless, the results have been interpreted cautiously, and confirmatory research using more rigorous study designs is recommended to validate these findings.

The classification and documentation of restrictive practice incidents probably vary across countries and legislative frameworks: for example, countries with a legal obligation to report tend to exhibit higher compliance rates than those without such a requirement. Sensitivity analyses accounting for clustering of data within countries and regions showed effects for selected scenarios, highlighting the need for more detailed regional comparisons of how various restrictive practice scenarios are classified and reported across different settings and jurisdictions. Further research is also encouraged to examine how factors such as national policies, cultural norms, staffing levels, professional training, workload and other systemic barriers influence HCPs’ ability to accurately and consistently classify and document restrictive practice incidents.

## Supporting information

10.1192/bjo.2026.11040.sm001 Belayneh et al. supplementary material 1 Belayneh et al. supplementary material

10.1192/bjo.2026.11040.sm002 Belayneh et al. supplementary material 2 Belayneh et al. supplementary material

10.1192/bjo.2026.11040.sm003 Belayneh et al. supplementary material 3 Belayneh et al. supplementary material

10.1192/bjo.2026.11040.sm004 Belayneh et al. supplementary material 4 Belayneh et al. supplementary material

## Data Availability

The data that support the findings of this study are available from the corresponding author, Z.B., upon request by email.

## Supplementary material

The supplementary material is available online at https://doi.org/10.1192/bjo.2026.11040
