# Supplementary material for: Discrepancies in classification and reporting of restrictive practices (restraints, seclusion and other coercive measures) in mental health services: multi-scenario analysis of an international survey
Source: BJPsych Open. 2026 May 11;12(3):e131. doi: 10.1192/bjo.2026.11040 (PMC13169055; doi:10.1192/bjo.2026.11040)
Supplement: Belayneh et al. supplementary material 1 — Belayneh et al. supplementary material [file S2056472426110400sup001.docx]

**Supplementary 1: A comprehensive list of contexts (n=23) identified through the co-design process and case scenarios reflecting these contexts (n=81)**

| Issue/context 1: Health practitioner’s intention / desired outcome of the action | |
| --- | --- |
| 1 | A nurse forcefully confines a person in a locked room to prevent suicide. |
| 2 | A nurse forcefully confines a person in a locked room for clinical observation. |
| 3 | A nurse forcefully confines a person in a locked room to maintain the person’s safety. |
| 4 | A nurse forcefully confines a person in a locked room to minimize the risk of harm to others. |
| 5 | A nurse forcefully confines a person in a locked room to minimize the risk of self-harm through cutting of body parts. |
| 6 | A nurse forcefully confines a person in a locked room to punish the person’s behaviour. |
| Issue/context 2: Who applies the action | |
| 7 | The person was displaying fearful behaviour by kicking the wall of his room. To minimize the risk of self-harm, **a staff member** firmly held the person's arm. |
| 8 | The person was displaying fearful behavior by kicking the wall of his room. To minimize the risk of self-harm, a security person (guard) firmly held the person's arm. |
| 9 | The person was displaying fearful behavior by kicking the wall of his room. To minimize the risk of self-harm, a peer worker firmly held the person's arm. |
| Issue/context 3: Persons’ ability being restricted (what the persons can no longer does during the episode of the action) | |
| 10 | A nurse prevents the person **from receiving visits from family, friends, or loved ones.** |
| 11 | A nurse restricts a person from **accessing social events without supervision from others.** |
| 12 | A nurse prevents the person from **accessing mobile phones and the internet.** |
| 13 | A nurse prevents the person from **being able to move at all.** |
| 14 | A nurse prevents the person from **leaving/exiting a designated area or space.** |
| 15 | A nurse prevents a person from **accessing hazardous items such as chemicals stored in wards** |
| Issue/context 4: Legal frameworks governing the implementation of the action | |
| 16 | A staff member prevents a male person from entering an area where female people commonly reside. The staff member was **fully aware that this area is legally designated exclusively for females.** |
| 17 | A staff member prevents a male person from entering an area where females commonly reside. The staff member was fully aware that this area is **not legally designated exclusively for females.** |
| 18 | A staff member prevents a male person from entering an area where females commonly reside. However, the staff member was **uncertain whether this area is legally designated exclusively for females.** |
| Issue/context 5: Number of restraint points applied on the person’s body parts | |
| 19 | The staff member applies chain restraints by securing **one of the** person**'s wrists** to the bed. |
| 20 | The staff member applies chain restraints by securing **both of the** person**’s wrists** to the bed. |
| 21 | The staff member applies chain restraints by securing **both** person**’s wrists and ankles** to the bed. |
| Issue/context 6: Level of consent | |
| 22 | The staff discusses the use of mechanical restraints with a person, resulting in the person consenting to the action. Then, the staff applies mechanical restraints to the person. |
| 23 | The staff discusses the use of mechanical restraints with a person, resulting in **the** person **refusing consent** to the action. Then, the staff applies mechanical restraints **against the** person**’s consent.** |
| 24 | The staff discusses the use of mechanical restraints with a person, but the person **refuses to give consent. Subsequently, staff engage in a discussion with the** person**'s family member and the family member granted consent.** The staff then applies mechanical restraints to the person. |
| 25 | The staff applies mechanical restraints to a person **without making efforts to obtain consent** from either the person or family member/caregivers. |
| 26 | The staff applies mechanical restraints to the person **without making efforts to obtain consent f**rom the person given the person’s **known cognitive impairments.** |
| Issue/context 7: Trial of less restrictive alternatives before initiating the action | |
| 27 | The medical team uses mechanical devices to restrain a person who is displaying fearful behaviour. The team **believe that this is the only option to achieve the desired outcome for that case before trying other alternative measures.** |
| 28 | The medical team uses mechanical devices to restrain a person who is displaying fearful behaviour. **The team initially tried sedative medications and found that sedatives were ineffective in that case.** |
| 29 | The medical team uses mechanical devices to restrain a person who is displaying fearful behaviour. **The team initially tried sedative medications and found that sedatives had some clinical benefit, but the desired clinical outcome was not fully achieved in that case.** |
| Issue/context 8: Door locking (locked doors versus open doors) | |
| 30 | Person is led to a single room **with the door being locked** to prevent the risk of self-harm. |
| 31 | Person is led to a single room with the **door left unlocked** to prevent the risk of self-harm. |
| 32 | Person is led to a single room with the **door left unlocked intermittently** to prevent the risk of self-harm. |
| Issue/context 9: Door locking (which door is being locked?) | |
| 33 | A nurse locks the **individual person's room door** to prevent them from escaping the hospital. |
| 34 | A nurse locks the **ward door** to prevent a person from escaping the hospital. |
| 35 | A nurse locks **the gate doors of an area enclosed by artificial barriers** to prevent a person from escaping the hospital. |
| Issue/context 10: Level of authority for the approval of the action | |
| 36 | A nurse administered sedative medication to a person who is disturbing the ward environment due to fearful behaviour**, with approval from a psychiatrist.** |
| 37 | A nurse administered sedative medication to a person who is disturbing the ward environment due to fearful behaviour, **with approval from a trained nurse practitioner.** |
| 38 | A nurse administered sedative medication to a person who is disturbing the ward environment due to fearful behaviour, **with approval from a trained general medical practitioner.** |
| 39 | A nurse administers sedative medication to a person who is disturbing the ward environment due to fearful behaviour, **without approval from any other authorities.** |
| Issue/context 11: Evidence supporting the necessity of the action | |
| 40 | A health practitioner forcefully confines a person to a seclusion room **after witnessing the person attempting** to physically harm others. |
| 41 | A health practitioner forcefully confines a person to a seclusion room **after overhearing the person expressing an intention** to physically harm others. |
| 42 | A health practitioner forcefully confines a person to a seclusion room **based on a report received from the person’s family caregivers, stating that the person intends to** physically harm others. |
| 43 | A health practitioner forcefully confines a person to a seclusion room **based on the person's previous history of physically harming others, but currently no identified intentions to harm others.** |
| Issue/context 12: Outcome of the action | |
| 44 | A person refuses to take the prescribed medication. Then, two staff members hold the person onto a bed to facilitate the administration of the medication. **The medication has been safely administered.** |
| 45 | A person refuses to take the prescribed medication. Then, two staff members hold the person onto a bed to facilitate the administration of the medication. **The health practitioner is unable to administer the medication due to the person’s uncontrollable behaviour.** |
| 46 | A person refuses to take the prescribed medication. Then, two staff members hold the person onto a bed to facilitate the administration of the medication. **Despite the medication being administered, the person sustains a needle injury during the process.** |
| Issue/context 13: Duration of a single episode of the action | |
| 47 | The medical team applies mechanical restraints to safely administer injectable medications to a person who exhibits fearful behaviour during hospital admission. The team **releases the mechanical restraints immediately** after administering the injection. |
| 48 | The medical team applies mechanical restraints to safely administer injectable medications to a person who exhibits fearful behaviour during hospital admission. The team decides to restrain the person for one hour after administering the injection. |
| 49 | The medical team applies mechanical restraints to safely administer injectable medications to a person who exhibits fearful behaviour during hospital admission. **The team decides to keep the person restrained for the entire day after administering the injection.** |
| Issue/context 14: Risk assessments | |
| 50 | A health practitioner is using belts to secure a person's arms and legs to the bed as a safety measure to prevent self-harm. This action is being taken **based on the risk assessment that indicates a higher risk of danger** for this person. |
| 51 | A health practitioner is using belts to secure a person's arms and legs to the bed as a safety measure to prevent self-harm. This action is being taken **based on the risk assessment that indicates a lower risk of danger** for this person. |
| 52 | A health practitioner is using belts to secure a person's arms and legs to the bed as a safety measure to prevent self-harm. This action is being taken **even though the risk assessment does not indicate any risk danger** for this person. |
| 53 | A health practitioner is using belts to secure a person's arms and legs to the bed as a safety measure to prevent self-harm. This action is being taken **without conducting a risk assessment** for this person. |
| Issues/context 15: Infliction of pain and person’s ability to easily control the action | |
| 54 | A staff member holds the person’s hand, and the person could release themselves from the staff’s hands if they want to without difficulty. No pain is involved. |
| 55 | A staff member holds the person’s hand, but the person cannot release themselves from the staff’s hands if they want to without difficulty. No pain is involved. |
| 56 | A staff member holds the person’s hand, but the person cannot release themselves from the staff’s hands if they wanted to without difficulty. Pain is involved. |
| Issue/context 16: Verbal orders/emotional restraint | |
| 57 | The nurse threatens the person with mechanical restraint **unless the person agrees to take the prescribed medication as directed.** |
| 58 | **The nurse promises to bring the person a soft drink if the person stays in the room** and **agrees to take the prescribed medication as directed.** |
| Issue/context 17: Timing when the action takes place | |
| 59 | A person is kept under mechanical restraint **upon arrival at the hospital** to ensure safety. |
| 60 | A person is kept under mechanical restraint during transportation from home to the hospital by police to ensure safety. |
| 61 | A person was kept under mechanical restraint **while being transferred between wards** to ensure safety. |
| 62 | A person is kept under mechanical restraint **until additional staff members arrive during a staff change-over** to ensure safety. |
| Issue/context 18: Shift type | |
| 63 | A staff member applies mechanical restraint to the person during a PM shift. |
| 64 | A staff member applies mechanical restraint to the person during an AM shift. |
| Issue/context 19: Availability of other individuals during seclusion episodes | |
| 65 | A person is securely locked **alone** in a room. |
| 66 | A person is securely locked in a room **while a staff member is present in the room with the person.** |
| 67 | A person is securely locked in a room **while a peer worker is present** in the room with the person. |
| 68 | A person is securely locked in a room **with a group of persons.** |
| 69 | A person is securely locked **alone in a room, while a staff member periodically monitors them using a security camera.** |
| 70 | A person is securely locked **alone in a room, while a staff member periodically monitors them through windows.** |
| Issue/context 20: Level of service facilities available in an isolation room | |
| 71 | An individual is kept in a closed, **empty room without furniture, windows, light, or toilets** to effectively manage agitated behaviour. |
| 72 | An individual is kept in a closed room that is **sparsely furnished but has no windows or toilets** to effectively manage agitated behaviour. |
| 73 | An individual is kept in a closed room that is **fully furnished with windows, light, and toilets t**o effectively manage agitated behaviour. |
| Issue/context 21: Adherence to the hospital’s policy/guideline | |
| 74 | A nurse administers sleep-inducing medication to a person. **The hospital has approved the use of this medication.** |
| 75 | A nurse administers sleep inducing medication to a person**.** T**his medication has not been approved for use in the hospital. However, the nurse decides to administer the medication to achieve an immediate response.** |
| 76 | A nurse administers sleep-inducing medication to a person. However, it is unclear whether t**his medication has been approved for use in the hospital. The nurse decides to administer the medication to achieve an immediate response.** |
| Issue/context 22: Dosage and frequency of medications | |
| 77 | A nurse administers sleep-inducing medication to a person. **Although the hospital has approved the use of this medication, the nurse increases the amount (dosage) of the medication to achieve an immediate response.** |
| 78 | A nurse administers sleep-inducing medication to a person. **Although the hospital has approved the use of this medication, the nurse administers the medication more frequently than the hospital’s standard to achieve an immediate response.** |
| Issue/context 23: Person position during the implementation of the actions | |
| 79 | A nurse applies mechanical restraints to a person by keeping the person **lying flat on their back, with their face and torso facing upwards (supine position).** |
| 80 | A nurse applies mechanical restraints to a person by keeping the person **lying flat on their stomach, with their face and torso facing downwards (prone position).** |
| 81 | A nurse applies mechanical restraints to a person by keeping them lying flat on their side, with their face and torso facing forward (lateral position). |

Note: The underlined statements in the descriptions of each case scenario represent parallel descriptions of the same context, with varying levels and options considered for comparison purpose.
