# Supplementary material for: Discrepancies in classification and reporting of restrictive practices (restraints, seclusion and other coercive measures) in mental health services: multi-scenario analysis of an international survey
Source: BJPsych Open. 2026 May 11;12(3):e131. doi: 10.1192/bjo.2026.11040 (PMC13169055; doi:10.1192/bjo.2026.11040)
Supplement: Belayneh et al. supplementary material 2 — Belayneh et al. supplementary material [file S2056472426110400sup002.pdf]

English (United Kingdom)

Introduction

Q1.

Explanatory statement and consent form

Project ID: 42246

Project Title: Classification and reporting of restrictive care practices

Chief Investigator's Name: Professor Terry Haines

School of Primary and Allied Health Care

Phone: +61 3 9902 9409, Email: terry.haines@monash.edu

Student's Name: Zelalem Belayneh Muluneh

School of Primary and Allied Health Care

Phone: +61456316020 Email: zelalem.muluneh@monash.edu

We invite you to participate in this survey because of your professional expertise or lived experiences in the field of mental health. This study is part of Zelalem's Ph.D. thesis being undertaken at Monash University. Please read this **Explanatory Statement** in full before deciding whether to participate in this research. If you would like further information regarding any aspect of this project, please contact the researchers via the phone numbers or email addresses listed above.

What does the research involve?

The objective of this study is to compare how individuals from different regions around the world classify and report restrictive care practices in adult mental health inpatient settings. **Restrictive care practices** refer to any actions or measures that have the effect of restricting the rights, freedom/choice or free movement of a care recipient. These practices include physical restraint, mechanical restraint, chemical restraint, seclusion, involuntary admission, or other coercive measures.

This survey is aimed at four groups of people:

1. People who have previously worked or are currently working as a **health professional** in an adult, mental health, inpatient setting.
2. People who have experience conducting **research** in the mental health field.
3. People who have **previously** received care in an adult, mental health, inpatient setting.
4. People who have **previously** provided informal care/support for a family member, friend, relative, or others who have been admitted to adult, mental health, inpatient facilities.
    - People under the age of 18 are **NOT** eligible to participate.
    - People who are **currently receiving care or have received care in the past six months in adult mental health inpatient settings** are NOT eligible to participate
    - People who are currently providing **informal care or support for a family member, friends, relatives, or other** individuals admitted to adult, mental health, inpatient settings are **NOT eligible** to participate

Consent to participate in the project and withdrawing from the research

Participation in this survey is voluntary. You have the option to accept or decline this invitation, and to withdraw from the study at any stage if you wish to do so. Data collection will be complete

when you click the "submit" button and partial survey completion data will not be used.

Possible benefits and risks to participants

We do not anticipate that you will experience any direct benefit through participation in this study. However, your participation will contribute to the development of programs designed to enhance the quality of mental healthcare. The survey includes questions on sensitive topics. Participants in this research will be asked to review case scenarios describing what could potentially be considered a restrictive care practice. Reviewing these case scenarios may evoke unpleasant memories and cause discomfort. However, we are seeking your perspectives on how these case scenarios should be classified and whether they should be reported. You will not be required to disclose any personal experiences related to the use of restrictive care practices. Please consult your doctor, local health professionals or other support systems if the survey has raised any distress. You can stop your participation in this study at any stage if you feel uncomfortable.

Confidentiality

This study uses an anonymous online questionnaire via the Qualtrics platform. All data will be collected in a de-identified form and no one, not even the researchers, will have the ability to identify you from your answers. Access to the data will be restricted solely to the research team.

Storage of data

All the collected data will be securely stored on a password-protected Monash University device and will remain confidential. Data will be stored for a minimum of 5 years after the completion of the project according to the Monash University data management policy. If the collected data are required for future research, de-identified and aggregated data will be shared upon request with the chief investigator. After 5 years, the data will be destroyed in line with the Monash University data destruction procedures.

Results

Our results will be shared publicly through conference-presentations and journal publications. Manuscripts will be incorporated into Zelalem's PhD thesis (one of the research team members). A plain language summary will be presented to relevant stakeholders. If you wish to receive a summary of project

findings or if you have any questions, please contact us using the emails provided above.

Complaints

Should you have any concerns or complaints about the conduct of this research, you are welcome to contact the Executive Officer, Monash University Human Research Ethics Committe (MUHREC):

Executive Officer, Office of Research Ethics and Integrity
Room 111, Building 3e
26 Sports Walk, Clayton Campus
Research Office Monash University VIC 3800
Tel: +61 3 9905 2052, Email: muhrec@monash.edu, Fax: +61 3 9905 3831

Consent statement

Please respond to the consent statement options below. You cannot access survey questions without responding to this question. By clicking the "Yes" option below, you acknowledge that your participation in the study is voluntary, that you are over the age of 18 years, and that you are aware that you may choose to terminate your participation in the study at any time and for any reason. The survey questions will automatically become available once you agree to participate and select the "Yes" option. If you select the "No" option, the survey will be terminated for you.

Would you like to participate in this study?

- ○ **Yes**, I understand the project and voluntarily agree to participate in this study.
- ○ **No**, I have chosen not to participate in this survey (the survey will end for you if you select this option).

Q2.

Eligibility Assessment

Which of the following groups of individuals best describes yourself?

- ◯ People who have **previously** worked or are **currently** working as a health professional in an adult, mental health, inpatient settings.
- ◯ People who have experience **conducting researc**h in the mental health field.
- ◯ People who have **previously** received care in an adult, mental health, inpatient settings.
- ◯ People who have **previously** provided informal care or support for a family member, friend, relative or others who have been admitted to adult mental health, inpatient settings.
- ◯ I **do not** have any of the above experiences (If you select this option, you are not eligible to participate in this study and the survey will be terminated for you).

Q3.

Section One: Socio-demographic Characteristics Questions

This section has questions relating to your socio-demographic characteristics. Please read each question carefully and respond accordingly.

What is you gender identity

- ◯ Male
- ◯ Female
- ◯ Non-binary/gender diverse
- ◯ My gender identity is not listed

- Preferred not to say

Q4. Please select your age category.

- 18 - 30 years
- 31 - 40 years
- 41 - 50 years
- 51 - 60 years
- 61 - 70 years
- 71 years and older

Q5. Please select the highest level of educational qualification you have completed.

- Primary School
- Secondary School
- Diploma
- Degree
- Masters
- Doctoral degree/PhD
- Others

Q6. Please select the country where you live from the dropdown options provided below.

Section Two: Exposure and Level of Experience with Mental Health

Q7.

Section Two: Exposure and Level of Experience in the Mental Health Sector

This section contains questions focusing on your professional background and /or experience in adult mental health settings, either as a **healthcare professional**, **a recipient of care**, **an informal caregiver** or **a researcher** in the mental health field.

Q8. Please select your professional discipline?

- ○ Medical doctor/Specialist
- ○ Nursing general
- ○ Psychiatric nursing
- ○ Public health
- ○ Psychology
- ○ Social work/Sociology
- ○ Occupational therapy
- ○ Others

Q9. Which shifts do you typically work in adult, mental health, inpatient settings? (Please select as many as apply)

- ☐ Morning shift
- ☐ Afternoon shift
- ☐ Night shift

Q10. What is the number of adult, mental health, inpatient beds normally available in the facility where you are currently working, or if you are not currently working, where you last worked?

- ○ Less than 10 beds
- ○ 10 to 29 beds
- ○ 30 to 49 beds
- ○ 50 to 99 beds
- ○ 100 or more beds

Q11. Which category corresponds to the number of years you have worked in adult, mental health, inpatient settings?

- ○ Less than 1 year

- 1 to 5 years
- 6 to 10 years
- More than 10 years

Q12. Does your adult mental health inpatient facility have an incident reporting system in place that can be used to record instances of restrictive care practices?

- Yes
- No
- Unsure

Q13.
Does your adult mental health inpatient facility have any policies or protocols that describe which scenarios should be **classified** as restrictive care practices?

- Yes
- No
- Unsure

Q14. Does your adult mental health inpatient facility have any policies or protocols that guide which scenarios

should be **recorded** as restrictive care practices in the hospital's reporting system?

- ○ Yes
- ○ No
- ○ Unsure

Q15. How long since you completed your PhD?

- ○ More than 10 years
- ○ 6-10 years
- ○ 0-5 years
- ○ I am currently undertaking a PhD
- ○ I have not completed a PhD and am not currently enrolled

Q16. Do you have any previous or current experience working as a clinician in adult, mental health, inpatient settings?

- ○ Yes, I am currently working.
- ○ Yes, I did in the past.
- ○ No, I do not have this experience.

Q17. Did you witness any restrictive care practices being used during your most recent stay within the adult mental

health inpatient setting while receiving care?

- ◯ Yes
- ◯ No

Q18. Did you feel distressed by this?

- ◯ Yes
- ◯ No

Q19. We encourage you to take a break or stop at any time and seek support if you do feel that this distress has been provoked by completing this survey.

Q20. What is the relation to you of the person who you most recently provided informal care to or support for while they were cared for in an adult, mental health, inpatient setting?

- ◯ My parent
- ◯ My spouse
- ◯ My son/daughter
- ◯ My sibling
- ◯ My other relative

- ◯ My close friend
- ◯ Other

Q21. Did you witness any restrictive care practices being used during the most recent stay of the person who you provided informal care to or support for?

- ◯ Yes
- ◯ No

Q22. Did you feel distressed by this

- ◯ Yes
- ◯ No

Q23. We encourage you to take a break or stop at any time and seek support if you do feel that this distress has been provoked by completing this survey.

Section Three: Classification and reporting of case scenario descriptions

Q24.

Section Three: Classification and Reporting of Case Scenarios

In this section, we will ask you questions about whether you think different scenarios should be **classified** as restrictive care practices or not, and whether they should be **recorded** in the hospital's reporting system.
For each scenario, we want you to consider that no prior attempts have been made to use a less restrictive approach with the patient unless this is specifically mentioned in the scenario.

Please carefully read the following lists of case scenarios and respond to the subsequent questions for each scenario.

Q25.

Case Scenario 1: A nurse forcefully confines a person in a locked room **to minimize the risk of harm to others.**

	Definitely yes	Pr
Do **you think** that this scenario **describes** a restrictive care practice?	○	

	Definitely yes	Pr
Is this action **classified** as a restrictive care practice within the mental health inpatient ward **where you work/have worked**?	○	
Do **you think** that this action should be **recorded** as a restrictive care practice in the hospital's reporting system?	○	
Do you think this action would be **documented** as a restrictive care practice in the hospital's reporting system by **other clinicians where you work/have worked?**	○	

Q26. How frequently have you observed this action being used in the adult mental health inpatient facility where you work/have most recently worked?

Never been observed	Once per year	Once per month	Once per week	Once or more per day	I do not wish to respond to this question
○	○	○	○	○	○

Q27. How frequently have you observed this action being used in the adult mental health inpatient facility that you have experienced?

Never been observed	Once per year	Once per month	Once per week	Once or more per day	I do not wish to respond to this question
○	○	○	○	○	○

Q28.

Case Scenario 2: A nurse forcefully confines a person in a locked room **to minimize the risk of self-harm by cutting body parts.**

	Definitely yes	Pr
Do **you think** that this scenario **describes** a restrictive care practice?	○	
Is this action **classified** as a restrictive care practice within the mental health inpatient ward **where you work/have worked**?	○	
Do **you think** that this action should be **recorded** as a restrictive care practice in the hospital's reporting system?	○	
Do you think this action would be **documented** as a restrictive care practice in the hospital's reporting system by **other clinicians where you work/have worked?**	○	

Q29. How frequently have you observed this action being used in the adult mental health inpatient facility where you work/have most recently worked?

Never been observed	Once per year	Once per month	Once per week	Once or more per day	I do not wish to respond to this question
○	○	○	○	○	○

Q30. How frequently have you observed this action being used in the adult mental health inpatient facility that you

have experienced?

Never been observed	Once per year	Once per month	Once per week	Once or more per day	I do not wish to respond to this question
○	○	○	○	○	○

Q31.

Case Scenario 3: A nurse forcefully confines a person in a locked room **to minimize the risk of suicide.**

	Definitely yes	Pr
Do **you think** that this scenario **describes** a restrictive care practice?	○	
Is this action **classified** as a restrictive care practice within the mental health inpatient ward **where you work/have worked**?	○	
Do **you think** that this action should be **recorded** as a restrictive care practice in the hospital's reporting system?	○	
Do you think this action would be **documented** as a restrictive care practice in the hospital's reporting system by **other clinicians where you work/have worked?**	○	

Q32. How frequently have you observed this action being used in the adult mental health inpatient facility where you work/have most recently worked?

Never been observed	Once per year	Once per month	Once per week	Once or more per day	I do not wish to respond to this question
○	○	○	○	○	○

Q33. How frequently have you observed this action being used in the adult mental health inpatient facility that you have experienced?

Never been observed	Once per year	Once per month	Once per week	Once or more per day	I do not wish to respond to this question
○	○	○	○	○	○

Q34.

Case Scenario 4: A person refuses to take prescribed medication so two nurses hold the person onto a bed to facilitate medication adminstration. **The medication is safely administered.**

	Definitely yes	Pr
Do **you think** that this scenario **describes** a restrictive care practice?	○	
Is this action **classified** as a restrictive care practice within the mental health inpatient ward **where you work/have worked**?	○	
Do **you think** that this action should be **recorded** as a restrictive care practice in the hospital's reporting system?	○	

	Definitely yes	Pr
Do you think this action would be **documented** as a restrictive care practice in the hospital's reporting system by **other clinicians where you work/have worked?**	○	

Q35. How frequently have you observed this action being used in the adult mental health inpatient facility where you work/have most recently worked?

Never been observed	Once per year	Once per month	Once per week	Once or more per day	I do not wish to respond to this question
○	○	○	○	○	○

Q36. How frequently have you observed this action being used in the adult mental health inpatient facility that you have experienced?

Never been observed	Once per year	Once per month	Once per week	Once or more per day	I do not wish to respond to this question
○	○	○	○	○	○

Q37.

Case Scenario 5. A person refuses to take prescribed medication so two nurses hold the person onto a bed to

facilitate administration of the medication. **The medication is administered but the patient sustains a needle injury during the process.**

	Definitely yes	Pr
Do **you think** that this scenario **describes** a restrictive care practice?	○	
Is this action **classified** as a restrictive care practice within the mental health inpatient ward **where you work/have worked**?	○	
Do **you think** that this action should be **recorded** as a restrictive care practice in the hospital's reporting system?	○	
Do you think this action would be **documented** as a restrictive care practice in the hospital's reporting system by **other clinicians where you work/have worked?**	○	

Q38. How frequently have you observed this action being used in the adult mental health inpatient facility where you work/have most recently worked?

Never been observed	Once per year	Once per month	Once per week	Once or more per day	I do not wish to respond to this question
○	○	○	○	○	○

Q39. How frequently have you observed this action being used in the adult mental health inpatient facility that you have experienced?

Never been observed	Once per year	Once per month	Once per week	Once or more per day	I do not wish to respond to this question
○	○	○	○	○	○

Q40.

Case Scenario 6: The person is displaying fearful behavior by punching the walls of their room. To minimize the risk of self-harm, **a security person (guard)** firmly holds the person's arm.

	Definitely yes	Pr
Do **you think** that this scenario **describes** a restrictive care practice?	○	
Is this action **classified** as a restrictive care practice within the mental health inpatient ward **where you work/have worked**?	○	
Do **you think** that this action should be **recorded** as a restrictive care practice in the hospital's reporting system?	○	
Do you think this action would be **documented** as a restrictive care practice in the hospital's reporting system by **other clinicians where you work/have worked?**	○	

Q41. How frequently have you observed this action being used in the adult mental health inpatient facility where you work/have most recently worked?

Never been observed	Once per year	Once per month	Once per week	Once or more per day	I do not wish to respond to this question
○	○	○	○	○	○

Q42. How frequently have you observed this action being used in the adult mental health inpatient facility that you have experienced?

Never been observed	Once per year	Once per month	Once per week	Once or more per day	I do not wish to respond to this question
○	○	○	○	○	○

Q43.

Case Scenario 7: The person is displaying fearful behavior by punching the wall of their room. To minimize the risk of self-harm, **a nurse** firmly holds the person's arm

	Definitely yes	Pr
Do **you think** that this scenario **describes** a restrictive care practice?	○	
Is this action **classified** as a restrictive care practice within the mental health inpatient ward **where you work/have worked**?	○	
Do **you think** that this action should be **recorded** as a restrictive care practice in the hospital's reporting system?	○	

	Definitely yes	Pr
Do you think this action would be **documented** as a restrictive care practice in the hospital's reporting system by **other clinicians where you work/have worked?**	○	

Q44. How frequently have you observed this action being used in the adult mental health inpatient facility where you work/have most recently worked?

Never been observed	Once per year	Once per month	Once per week	Once or more per day	I do not wish to respond to this question
○	○	○	○	○	○

Q45. How frequently have you observed this action being used in the adult mental health inpatient facility that you have experienced?

Never been observed	Once per year	Once per month	Once per week	Once or more per day	I do not wish to respond to this question
○	○	○	○	○	○

Q46.

Case Scenario 8: A nurse applies mechanical restraints to a person by keeping the person **lying flat on their stomach,**

with their face and chest pointing downwards (prone position).

	Definitely yes	Pr
Do **you think** that this scenario **describes** a restrictive care practice?	○	
Is this action **classified** as a restrictive care practice within the mental health inpatient ward **where you work/have worked**?	○	
Do **you think** that this action should be **recorded** as a restrictive care practice in the hospital's reporting system?	○	
Do you think this action would be **documented** as a restrictive care practice in the hospital's reporting system by **other clinicians where you work/have worked?**	○	

Q47. How frequently have you observed this action being used in the adult mental health inpatient facility where you work/have most recently worked?

Never been observed	Once per year	Once per month	Once per week	Once or more per day	I do not wish to respond to this question
○	○	○	○	○	○

Q48. How frequently have you observed this action being used in the adult mental health inpatient facility that you have experienced?

Never been observed	Once per year	Once per month	Once per week	Once or more per day	I do not wish to respond to this question
○	○	○	○	○	○

Q49.

Case Scenario 9: A nurse applies mechanical restraints to a person by keeping the person **lying flat on their back, with their face and chest pointing upwards (supine position).**

	Definitely yes	Pr
Do **you think** that this scenario **describes** a restrictive care practice?	○	
Is this action **classified** as a restrictive care practice within the mental health inpatient ward **where you work/have worked**?	○	
Do **you think** that this action should be **recorded** as a restrictive care practice in the hospital's reporting system?	○	
Do you think this action would be **documented** as a restrictive care practice in the hospital's reporting system by **other clinicians where you work/have worked?**	○	

Q50. How frequently have you observed this action being used in the adult mental health inpatient facility where you work/have most recently worked?

Never been observed	Once per year	Once per month	Once per week	Once or more per day	I do not wish to respond to this question
○	○	○	○	○	○

Q51. How frequently have you observed this action being used in the adult mental health inpatient facility that you have experienced?

Never been observed	Once per year	Once per month	Once per week	Once or more per day	I do not wish to respond to this question
○	○	○	○	○	○

Q52.

Case Scenario 10: A nurse locks the **individual person's room** door to prevent them from escaping the hospital.

	Definitely yes	Pr
Do **you think** that this scenario **describes** a restrictive care practice?	○	
Is this action **classified** as a restrictive care practice within the mental health inpatient ward **where you work/have worked**?	○	
Do **you think** that this action should be **recorded** as a restrictive care practice in the hospital's reporting system?	○	
Do you think this action would be **documented** as a restrictive care practice in the hospital's reporting system by **other clinicians where you work/have worked?**	○	

Q53. How frequently have you observed this action being used in the adult mental health inpatient facility where you work/have most recently worked?

Never been observed	Once per year	Once per month	Once per week	Once or more per day	I do not wish to respond to this question
○	○	○	○	○	○

Q54. How frequently have you observed this action being used in the adult mental health inpatient facility that you have experienced?

Never been observed	Once per year	Once per month	Once per week	Once or more per day	I do not wish to respond to this question
○	○	○	○	○	○

Q55.

Case Scenario 11: A nurse locks **the whole ward door** to prevent a person from escaping the hospital.

	Definitely yes	Pr
Do **you think** that this scenario **describes** a restrictive care practice?	○	
Is this action **classified** as a restrictive care practice within the mental health inpatient ward **where you work/have worked**?	○	
Do **you think** that this action should be **recorded** as a restrictive care practice in the hospital's reporting system?	○	

	Definitely yes	Pr
Do you think this action would be **documented** as a restrictive care practice in the hospital's reporting system by **other clinicians where you work/have worked?**	◯	

Q56. How frequently have you observed this action being used in the adult mental health inpatient facility where you work/have most recently worked?

Never been observed	Once per year	Once per month	Once per week	Once or more per day	I do not wish to respond to this question
◯	◯	◯	◯	◯	◯

Q57. How frequently have you observed this action being used in the adult mental health inpatient facility that you have experienced?

Never been observed	Once per year	Once per month	Once per week	Once or more per day	I do not wish to respond to this question
◯	◯	◯	◯	◯	◯

Q58.

Case Scenario 12: A person is kept under mechanical restraint **while being transferred between wards** to promote safety.

	Definitely yes	Pr
Do **you think** that this scenario **describes** a restrictive care practice?	○	
Is this action **classified** as a restrictive care practice within the mental health inpatient ward **where you work/have worked**?	○	
Do **you think** that this action should be **recorded** as a restrictive care practice in the hospital's reporting system?	○	
Do you think this action would be **documented** as a restrictive care practice in the hospital's reporting system by **other clinicians where you work/have worked?**	○	

Q59. How frequently have you observed this action being used in the adult mental health inpatient facility where you work/have most recently worked?

Never been observed	Once per year	Once per month	Once per week	Once or more per day	I do not wish to respond to this question
○	○	○	○	○	○

Q60. How frequently have you observed this action being used in the adult mental health inpatient facility that you have experienced?

Never been observed	Once per year	Once per month	Once per week	Once or more per day	I do not wish to respond to this question
○	○	○	○	○	○

Q61.

Case Scenario 13: A person is kept under mechanical restraint **when staff feel overloaded during busy days** to promote safety.

	Definitely yes	Pr
Do **you think** that this scenario **describes** a restrictive care practice?	○	
Is this action **classified** as a restrictive care practice within the mental health inpatient ward **where you work/have worked**?	○	
Do **you think** that this action should be **recorded** as a restrictive care practice in the hospital's reporting system?	○	
Do you think this action would be **documented** as a restrictive care practice in the hospital's reporting system by **other clinicians where you work/have worked?**	○	

Q62. How frequently have you observed this action being used in the adult mental health inpatient facility where you work/have most recently worked?

Never been observed	Once per year	Once per month	Once per week	Once or more per day	I do not wish to respond to this question
○	○	○	○	○	○

Q63. How frequently have you observed this action being used in the adult mental health inpatient facility that you have experienced?

Never been observed	Once per year	Once per month	Once per week	Once or more per day	I do not wish to respond to this question
○	○	○	○	○	○

Q64.

Case Scenario 14**:** Nurses discuss use of mechanical restraints with the person, but **the person refused to give consent to this action**. Then, the nurses apply mechanical restraints to the person.

	Definitely yes	Pr
Do **you think** that this scenario **describes** a restrictive care practice?	○	
Is this action **classified** as a restrictive care practice within the mental health inpatient ward **where you work/have worked**?	○	
Do **you think** that this action should be **recorded** as a restrictive care practice in the hospital's reporting system?	○	
Do you think this action would be **documented** as a restrictive care practice in the hospital's reporting system by **other clinicians where you work/have worked?**	○	

Q65. How frequently have you observed this action being used in the adult mental health inpatient facility where

you work/have most recently worked?

Never been observed	Once per year	Once per month	Once per week	Once or more per day	I do not wish to respond to this question
○	○	○	○	○	○

Q66. How frequently have you observed this action being used in the adult mental health inpatient facility that you have experienced?

Never been observed	Once per year	Once per month	Once per week	Once or more per day	I do not wish to respond to this question
○	○	○	○	○	○

Q67.

Case Scenario 15: Nurses discuss use of mechanical restraints with the person, but the **person refused to give consent to this action. These nurses later engaged in a discussion with the person's family member, and the family member granted consent**. The staff then applied mechanical restraints to the person.

	Definitely yes	Pr
Do **you think** that this scenario **describes** a restrictive care practice?	○	
Is this action **classified** as a restrictive care practice within the mental health inpatient ward **where you work/have worked**?	○	
Do **you think** that this action should be **recorded** as a restrictive care practice in the hospital's reporting system?	○	

	Definitely yes	Pr
Do you think this action would be **documented** as a restrictive care practice in the hospital's reporting system by **other clinicians where you work/have worked?**	○	

Q68. How frequently have you observed this action being used in the adult mental health inpatient facility where you work/have most recently worked?

Never been observed	Once per year	Once per month	Once per week	Once or more per day	I do not wish to respond to this question
○	○	○	○	○	○

Q69. How frequently have you observed this action being used in the adult mental health inpatient facility that you have experienced?

Never been observed	Once per year	Once per month	Once per week	Once or more per day	I do not wish to respond to this question
○	○	○	○	○	○

Q70.

Case Scenario 16: A nurse forcefully confines a person to a seclusion room **after witnessing the person attempting** to physically harm others.

	Definitely yes	Pr
Do **you think** that this scenario **describes** a restrictive care practice?	○	
Is this action **classified** as a restrictive care practice within the mental health inpatient ward **where you work/have worked**?	○	
Do **you think** that this action should be **recorded** as a restrictive care practice in the hospital's reporting system?	○	
Do you think this action would be **documented** as a restrictive care practice in the hospital's reporting system by **other clinicians where you work/have worked?**	○	

Q71. How frequently have you observed this action being used in the adult mental health inpatient facility where you work/have most recently worked?

Never been observed	Once per year	Once per month	Once per week	Once or more per day	I do not wish to respond to this question
○	○	○	○	○	○

Q72. How frequently have you observed this action being used in the adult mental health inpatient facility that you have experienced?

Never been observed	Once per year	Once per month	Once per week	Once or more per day	I do not wish to respond to this question
○	○	○	○	○	○

Q73.

Case Scenario 17**:** A nurse forcefully confines a person to a seclusion room **after overhearing the person verbally expressing an intention** to physically harm others.

	Definitely yes	Pr
Do **you think** that this scenario **describes** a restrictive care practice?	○	
Is this action **classified** as a restrictive care practice within the mental health inpatient ward **where you work/have worked**?	○	
Do **you think** that this action should be **recorded** as a restrictive care practice in the hospital's reporting system?	○	
Do you think this action would be **documented** as a restrictive care practice in the hospital's reporting system by **other clinicians where you work/have worked?**	○	

Q74. How frequently have you observed this action being used in the adult mental health inpatient facility where you work/have most recently worked?

Never been observed	Once per year	Once per month	Once per week	Once or more per day	I do not wish to respond to this question
○	○	○	○	○	○

Q75. How frequently have you observed this action being used in the adult mental health inpatient facility that you have experienced?

Never been observed	Once per year	Once per month	Once per week	Once or more per day	I do not wish to respond to this question
○	○	○	○	○	○

Q76.

Case Scenario 18: The medical team **applies** mechanical restraints to safely administer injectable medications to a person who exhibits fearful behaviors during hospital admission. **The team releases the mechanical restraints immediately** after administering the injection.

	Definitely yes	Pr
Do **you think** that this scenario **describes** a restrictive care practice?	○	
Is this action **classified** as a restrictive care practice within the mental health inpatient ward **where you work/have worked**?	○	
Do **you think** that this action should be **recorded** as a restrictive care practice in the hospital's reporting system?	○	
Do you think this action would be **documented** as a restrictive care practice in the hospital's reporting system by **other clinicians where you work/have worked?**	○	

Q77. How frequently have you observed this action being used in the adult mental health inpatient facility where you work/have most recently worked?

Never been observed	Once per year	Once per month	Once per week	Once or more per day	I do not wish to respond to this question
○	○	○	○	○	○

Q78. How frequently have you observed this action being used in the adult mental health inpatient facility that you have experienced?

Never been observed	Once per year	Once per month	Once per week	Once or more per day	I do not wish to respond to this question
○	○	○	○	○	○

Q79.

Case scenario 19: The medical team **applies** mechanical restraints to safely administer injectable medications to a person who exhibits fearful behaviors during hospital admission. **The team decided to keep the person restrained for one hour after administering the injection.**

	Definitely yes	Pr
Do **you think** that this scenario **describes** a restrictive care practice?	○	
Is this action **classified** as a restrictive care practice within the mental health inpatient ward **where you work/have worked**?	○	

	Definitely yes	Pr
Do **you think** that this action should be **recorded** as a restrictive care practice in the hospital’s reporting system?	○	
Do you think this action would be **documented** as a restrictive care practice in the hospital's reporting system by **other clinicians where you work/have worked?**	○	

Q80. How frequently have you observed this action being used in the adult mental health inpatient facility where you work/have most recently worked?

Never been observed	Once per year	Once per month	Once per week	Once or more per day	I do not wish to respond to this question
○	○	○	○	○	○

Q81. How frequently have you observed this action being used in the adult mental health inpatient facility that you have experienced?

Never been observed	Once per year	Once per month	Once per week	Once or more per day	I do not wish to respond to this question
○	○	○	○	○	○

Q82.

Case Scenario 20: The medical team t**threatens to use** mechanical restraint to safely administer injectable medications to a person who exhibits fearful behavior during hospital admission.

	Definitely yes	Pr
Do **you think** that this scenario **describes** a restrictive care practice?	◯	
Is this action **classified** as a restrictive care practice within the mental health inpatient ward **where you work/have worked**?	◯	
Do **you think** that this action should be **recorded** as a restrictive care practice in the hospital's reporting system?	◯	
Do you think this action would be **documented** as a restrictive care practice in the hospital's reporting system by **other clinicians where you work/have worked?**	◯	

Q83. How frequently have you observed this action being used in the adult mental health inpatient facility where you work/have most recently worked?

Never been observed	Once per year	Once per month	Once per week	Once or more per day	I do not wish to respond to this question
◯	◯	◯	◯	◯	◯

Q84. How frequently have you observed this action being used in the adult mental health inpatient facility that you have experienced?

Never been observed	Once per year	Once per month	Once per week	Once or more per day	I do not wish to respond to this question
○	○	○	○	○	○

Q85.

Case Scenario 21: A nurse uses belts to secure a person's arms and legs to the bed as a safety measure to prevent self-harm. This action is taken **based on the risk assessment that indicates a higher risk of danger** for this person.

	Definitely yes	Pr
Do **you think** that this scenario **describes** a restrictive care practice?	○	
Is this action **classified** as a restrictive care practice within the mental health inpatient ward **where you work/have worked**?	○	
Do **you think** that this action should be **recorded** as a restrictive care practice in the hospital's reporting system?	○	
Do you think this action would be **documented** as a restrictive care practice in the hospital's reporting system by **other clinicians where you work/have worked?**	○	

Q86. How frequently have you observed this action being used in the adult mental health inpatient facility where you work/have most recently worked?

Never been observed	Once per year	Once per month	Once per week	Once or more per day	I do not wish to respond to this question
○	○	○	○	○	○

Q87. How frequently have you observed this action being used in the adult mental health inpatient facility that you have experienced?

Never been observed	Once per year	Once per month	Once per week	Once or more per day	I do not wish to respond to this question
○	○	○	○	○	○

Q88.

Case scenario 22: A nurse uses belts to secure a person's arms and legs to the bed as a safety measure to prevent self-harm. This action is taken **without conducting a risk assessment** for this person.

	Definitely yes	Pr
Do **you think** that this scenario **describes** a restrictive care practice?	○	
Is this action **classified** as a restrictive care practice within the mental health inpatient ward **where you work/have worked**?	○	
Do **you think** that this action should be **recorded** as a restrictive care practice in the hospital's reporting system?	○	
Do you think this action would be **documented** as a restrictive care practice in the hospital's reporting system by **other clinicians where you work/have worked?**	○	

Q89. How frequently have you observed this action being used in the adult mental health inpatient facility where you work/have most recently worked?

Never been observed	Once per year	Once per month	Once per week	Once or more per day	I do not wish to respond to this question
○	○	○	○	○	○

Q90. How frequently have you observed this action being used in the adult mental health inpatient facility that you have experienced?

Never been observed	Once per year	Once per month	Once per week	Once or more per day	I do not wish to respond to this question
○	○	○	○	○	○

Sectio Four: Additional set of case scenarios

Q91.
Thank you for completing the first set of questions. You have now completed the **core item questions** of the survey. There is an additional set of questions with a similar number of case scenarios available.

You may complete as many of these additional questions as you like before submitting your response, or you can end the survey now

if you wish.

Would you like to proceed with more questions?

- ◯ Yes (If you select this option, you can access the additional set of questions by clicking the next button)
- ◯ No (If you select this option, your responses will be submitted and the survey will be ended for you)

Q92.

Section Four: Additional set of case scenarios

Thank you for your interest in answering an additional set of questions.
If you wish to end the survey before completing all the case scenarios, you may stop at any time, and your responses will be recorded automatically.

Case scenario 23: A person is led to a single room to prevent self-harm **and the door is locked.**

	Definitely yes	Pr
Do **you think** that this scenario **describes** a restrictive care practice?	◯	

	Definitely yes	Pr
Is this action **classified** as a restrictive care practice within the mental health inpatient ward **where you work/have worked**?	◯	
Do **you think** that this action should be **recorded** as a restrictive care practice in the hospital's reporting system?	◯	
Do you think this action would be **documented** as a restrictive care practice in the hospital's reporting system by **other clinicians where you work/have worked?**	◯	

Q93. How frequently have you observed this action being used in the adult mental health inpatient facility where you work/have most recently worked?

Never been observed	Once per year	Once per month	Once per week	Once or more per day	I do not wish to respond to this question
◯	◯	◯	◯	◯	◯

Q94. How frequently have you observed this action being used in the adult mental health inpatient facility that you have experienced?

Never been observed	Once per year	Once per month	Once per week	Once or more per day	I do not wish to respond to this question
◯	◯	◯	◯	◯	◯

Q95.

Case scenario 24: A person is led to a single room to prevent self-harm, **but the door is left unlocked.**

	Definitely yes	Pr
Do **you think** that this scenario **describes** a restrictive care practice?	○	
Is this action **classified** as a restrictive care practice within the mental health inpatient ward **where you work/have worked**?	○	
Do **you think** that this action should be **recorded** as a restrictive care practice in the hospital's reporting system?	○	
Do you think this action would be **documented** as a restrictive care practice in the hospital's reporting system by **other clinicians where you work/have worked?**	○	

Q96. How frequently have you observed this action being used in the adult mental health inpatient facility where you work/have most recently worked?

Never been observed	Once per year	Once per month	Once per week	Once or more per day	I do not wish to respond to this question
○	○	○	○	○	○

Q97. How frequently have you observed this action being used in the adult mental health inpatient facility that you

have experienced?

Never been observed	Once per year	Once per month	Once per week	Once or more per day	I do not wish to respond to this question
○	○	○	○	○	○

Q98.

Case scenario 25: The medical team uses mechanical devices to restrain a person who is displaying fearful behaviour. **The team initially tried sedative medications, but they were not effective.**

	Definitely yes	Pr
Do **you think** that this scenario **describes** a restrictive care practice?	○	
Is this action **classified** as a restrictive care practice within the mental health inpatient ward **where you work/have worked**?	○	
Do **you think** that this action should be **recorded** as a restrictive care practice in the hospital's reporting system?	○	
Do you think this action would be **documented** as a restrictive care practice in the hospital's reporting system by **other clinicians where you work/have worked?**	○	

Q99. How frequently have you observed this action being used in the adult mental health inpatient facility where you work/have most recently worked?

Never been observed	Once per year	Once per month	Once per week	Once or more per day	I do not wish to respond to this question
○	○	○	○	○	○

Q100. How frequently have you observed this action being used in the adult mental health inpatient facility that you have experienced?

Never been observed	Once per year	Once per month	Once per week	Once or more per day	I do not wish to respond to this question
○	○	○	○	○	○

Q101.
Case scenario 26: The medical team uses mechanical devices to restrain a person who is displaying fearful behaviour. The team **thinks that this is the only option to achieve the desired outcome, but they did not try other approaches first.**

	Definitely yes	Pr
Do **you think** that this scenario **describes** a restrictive care practice?	○	
Is this action **classified** as a restrictive care practice within the mental health inpatient ward **where you work/have worked**?	○	
Do **you think** that this action should be **recorded** as a restrictive care practice in the hospital's reporting system?	○	

	Definitely yes	Pr
Do you think this action would be **documented** as a restrictive care practice in the hospital's reporting system by **other clinicians where you work/have worked?**	◯	

Q102. How frequently have you observed this action being used in the adult mental health inpatient facility where you work/have most recently worked?

Never been observed	Once per year	Once per month	Once per week	Once or more per day	I do not wish to respond to this question
◯	◯	◯	◯	◯	◯

Q103. How frequently have you observed this action being used in the adult mental health inpatient facility that you have experienced?

Never been observed	Once per year	Once per month	Once per week	Once or more per day	I do not wish to respond to this question
◯	◯	◯	◯	◯	◯

Q104.

Case scenario 27: A nurse applies chain restraints by securing **both of the person's wrists and ankles** to a bed.

	Definitely yes	Pr
Do **you think** that this scenario **describes** a restrictive care practice?	○	
Is this action **classified** as a restrictive care practice within the mental health inpatient ward **where you work/have worked**?	○	
Do **you think** that this action should be **recorded** as a restrictive care practice in the hospital's reporting system?	○	
Do you think this action would be **documented** as a restrictive care practice in the hospital's reporting system by **other clinicians where you work/have worked?**	○	

Q105. How frequently have you observed this action being used in the adult mental health inpatient facility where you work/have most recently worked?

Never been observed	Once per year	Once per month	Once per week	Once or more per day	I do not wish to respond to this question
○	○	○	○	○	○

Q106. How frequently have you observed this action being used in the adult mental health inpatient facility that you have experienced?

Never been observed	Once per year	Once per month	Once per week	Once or more per day	I do not wish to respond to this question
○	○	○	○	○	○

Q107.

Case scenario 28: A nurse applies chain restraints by securing **one of the person's wrists and ankles** to a bed.

	Definitely yes	Pr
Do **you think** that this scenario **describes** a restrictive care practice?	○	
Is this action **classified** as a restrictive care practice within the mental health inpatient ward **where you work/have worked**?	○	
Do **you think** that this action should be **recorded** as a restrictive care practice in the hospital's reporting system?	○	
Do you think this action would be **documented** as a restrictive care practice in the hospital's reporting system by **other clinicians where you work/have worked?**	○	

Q108. How frequently have you observed this action being used in the adult mental health inpatient facility where you work/have most recently worked?

Never been observed	Once per year	Once per month	Once per week	Once or more per day	I do not wish to respond to this question
○	○	○	○	○	○

Q109. How frequently have you observed this action being used in the adult mental health inpatient facility that you have experienced?

Never been observed	Once per year	Once per month	Once per week	Once or more per day	I do not wish to respond to this question
○	○	○	○	○	○

Q110.

Case scenario 29: A nurse applies chain restraints by securing **one of the person's wrists** to a bed.

	Definitely yes	Pr
Do **you think** that this scenario **describes** a restrictive care practice?	○	
Is this action **classified** as a restrictive care practice within the mental health inpatient ward **where you work/have worked**?	○	
Do **you think** that this action should be **recorded** as a restrictive care practice in the hospital's reporting system?	○	
Do you think this action would be **documented** as a restrictive care practice in the hospital's reporting system by **other clinicians where you work/have worked?**	○	

Q111. How frequently have you observed this action being used in the adult mental health inpatient facility where you work/have most recently worked?

Never been observed	Once per year	Once per month	Once per week	Once or more per day	I do not wish to respond to this question
○	○	○	○	○	○

Q112. How frequently have you observed this action being used in the adult mental health inpatient facility that you have experienced?

Never been observed	Once per year	Once per month	Once per week	Once or more per day	I do not wish to respond to this question
○	○	○	○	○	○

Q113.

Case scenario 30: A nurse securely locks the door of the person's room as a safety measure **during the nighttime**.

	Definitely yes	Pr
Do **you think** that this scenario **describes** a restrictive care practice?	○	
Is this action **classified** as a restrictive care practice within the mental health inpatient ward **where you work/have worked**?	○	
Do **you think** that this action should be **recorded** as a restrictive care practice in the hospital's reporting system?	○	

	Definitely yes	Pr
Do you think this action would be **documented** as a restrictive care practice in the hospital's reporting system by **other clinicians where you work/have worked?**	◯	

Q114. How frequently have you observed this action being used in the adult mental health inpatient facility where you work/have most recently worked?

Never been observed	Once per year	Once per month	Once per week	Once or more per day	I do not wish to respond to this question
◯	◯	◯	◯	◯	◯

Q115. How frequently have you observed this action being used in the adult mental health inpatient facility that you have experienced?

Never been observed	Once per year	Once per month	Once per week	Once or more per day	I do not wish to respond to this question
◯	◯	◯	◯	◯	◯

Q116.

Case scenario 31: A nurse securely locks the door of the person's room as a safety measure **during the daytime.**

	Definitely yes	Pr
Do **you think** that this scenario **describes** a restrictive care practice?	○	
Is this action **classified** as a restrictive care practice within the mental health inpatient ward **where you work/have worked**?	○	
Do **you think** that this action should be **recorded** as a restrictive care practice in the hospital's reporting system?	○	
Do you think this action would be **documented** as a restrictive care practice in the hospital's reporting system by **other clinicians where you work/have worked?**	○	

Q117. How frequently have you observed this action being used in the adult mental health inpatient facility where you work/have most recently worked?

Never been observed	Once per year	Once per month	Once per week	Once or more per day	I do not wish to respond to this question
○	○	○	○	○	○

Q118. How frequently have you observed this action being used in the adult mental health inpatient facility that you have experienced?

Never been observed	Once per year	Once per month	Once per week	Once or more per day	I do not wish to respond to this question
○	○	○	○	○	○

Q119.

Case scenario 32: An individual was kept in a closed, **empty room without furniture, windows, light, or toilets** to manage agitated behaviour.

	Definitely yes	Pr
Do **you think** that this scenario **describes** a restrictive care practice?	○	
Is this action **classified** as a restrictive care practice within the mental health inpatient ward **where you work/have worked**?	○	
Do **you think** that this action should be **recorded** as a restrictive care practice in the hospital's reporting system?	○	
Do you think this action would be **documented** as a restrictive care practice in the hospital's reporting system by **other clinicians where you work/have worked?**	○	

Q120. How frequently have you observed this action being used in the adult mental health inpatient facility where you work/have most recently worked?

Never been observed	Once per year	Once per month	Once per week	Once or more per day	I do not wish to respond to this question
○	○	○	○	○	○

Q121. How frequently have you observed this action being used in the adult mental health inpatient facility that you have experienced?

Never been observed	Once per year	Once per month	Once per week	Once or more per day	I do not wish to respond to this question
○	○	○	○	○	○

Q122.
Case scenario 33: An individual is kept in a closed room that is **fully furnished with windows, light, and toilets** to manage agitated behaviour.

	Definitely yes	Pr
Do **you think** that this scenario **describes** a restrictive care practice?	○	
Is this action **classified** as a restrictive care practice within the mental health inpatient ward **where you work/have worked**?	○	
Do **you think** that this action should be **recorded** as a restrictive care practice in the hospital's reporting system?	○	
Do you think this action would be **documented** as a restrictive care practice in the hospital's reporting system by **other clinicians where you work/have worked?**	○	

Q123. How frequently have you observed this action being used in the adult mental health inpatient facility where

you work/have most recently worked?

Never been observed	Once per year	Once per month	Once per week	Once or more per day	I do not wish to respond to this question
○	○	○	○	○	○

Q124. How frequently have you observed this action being used in the adult mental health inpatient facility that you have experienced?

Never been observed	Once per year	Once per month	Once per week	Once or more per day	I do not wish to respond to this question
○	○	○	○	○	○

Q125.
Case scenario 34: A nurse administers sleep-inducing medication to a person suffering from insomnia. **Although the hospital has approved the use of this medication, the nurse increases the amount (dosage) of the medication to achieve an immediate clinical response**.

	Definitely yes	Pr
Do **you think** that this scenario **describes** a restrictive care practice?	○	
Is this action **classified** as a restrictive care practice within the mental health inpatient ward **where you work/have worked**?	○	
Do **you think** that this action should be **recorded** as a restrictive care practice in the hospital's reporting system?	○	

	Definitely yes	Pr
Do you think this action would be **documented** as a restrictive care practice in the hospital's reporting system by **other clinicians where you work/have worked?**	○	

Q126. How frequently have you observed this action being used in the adult mental health inpatient facility where you work/have most recently worked?

Never been observed	Once per year	Once per month	Once per week	Once or more per day	I do not wish to respond to this question
○	○	○	○	○	○

Q127. How frequently have you observed this action being used in the adult mental health inpatient facility that you have experienced?

Never been observed	Once per year	Once per month	Once per week	Once or more per day	I do not wish to respond to this question
○	○	○	○	○	○

Q128.

Case scenario 35: A nurse administers sleep-inducing medication to a person suffering from insomnia**. This medication is not approved for use in the hospital.**

However, the nurse decides to administer the medication to achieve an immediate clinical response.

	Definitely yes	Pr
Do **you think** that this scenario **describes** a restrictive care practice?	○	
Is this action **classified** as a restrictive care practice within the mental health inpatient ward **where you work/have worked**?	○	
Do **you think** that this action should be **recorded** as a restrictive care practice in the hospital's reporting system?	○	
Do you think this action would be **documented** as a restrictive care practice in the hospital's reporting system by **other clinicians where you work/have worked?**	○	

Q129. How frequently have you observed this action being used in the adult mental health inpatient facility where you work/have most recently worked?

Never been observed	Once per year	Once per month	Once per week	Once or more per day	I do not wish to respond to this question
○	○	○	○	○	○

Q130. How frequently have you observed this action being used in the adult mental health inpatient facility that you have experienced?

Never been observed	Once per year	Once per month	Once per week	Once or more per day	I do not wish to respond to this question
◯	◯	◯	◯	◯	◯

Q131.

Case scenario 36: A nurse administers sleep-inducing medication to a person suffering from insomnia. **The hospital has approved the use of this medication, and the nurse administers the prescribed dose according to the hospital's protocol.**

	Definitely yes	Pr
Do **you think** that this scenario **describes** a restrictive care practice?	◯	
Is this action **classified** as a restrictive care practice within the mental health inpatient ward **where you work/have worked**?	◯	
Do **you think** that this action should be **recorded** as a restrictive care practice in the hospital's reporting system?	◯	
Do you think this action would be **documented** as a restrictive care practice in the hospital's reporting system by **other clinicians where you work/have worked?**	◯	

Q132. How frequently have you observed this action being used in the adult mental health inpatient facility where you work/have most recently worked?

Never been observed	Once per year	Once per month	Once per week	Once or more per day	I do not wish to respond to this question
◯	◯	◯	◯	◯	◯

Q133. How frequently have you observed this action being used in the adult mental health inpatient facility that you have experienced?

Never been observed	Once per year	Once per month	Once per week	Once or more per day	I do not wish to respond to this question
○	○	○	○	○	○

Q134.

Case scenario 37: A nurse prevents the person from **leaving/exiting a designated area or space**.

	Definitely yes	Pr
Do **you think** that this scenario **describes** a restrictive care practice?	○	
Is this action **classified** as a restrictive care practice within the mental health inpatient ward **where you work/have worked**?	○	
Do **you think** that this action should be **recorded** as a restrictive care practice in the hospital's reporting system?	○	
Do you think this action would be **documented** as a restrictive care practice in the hospital's reporting system by **other clinicians where you work/have worked?**	○	

Q135. How frequently have you observed this action being used in the adult mental health inpatient facility where you work/have most recently worked?

Never been observed	Once per year	Once per month	Once per week	Once or more per day	I do not wish to respond to this question
○	○	○	○	○	○

Q136. How frequently have you observed this action being used in the adult mental health inpatient facility that you have experienced?

Never been observed	Once per year	Once per month	Once per week	Once or more per day	I do not wish to respond to this question
○	○	○	○	○	○

Q137.

Case scenario 38: A nurse prevents the person **from receiving visits from family, friends, or loved ones.**

	Definitely yes	Pr
Do **you think** that this scenario **describes** a restrictive care practice?	○	
Is this action **classified** as a restrictive care practice within the mental health inpatient ward **where you work/have worked**?	○	
Do **you think** that this action should be **recorded** as a restrictive care practice in the hospital's reporting system?	○	

	Definitely yes	Pr
Do you think this action would be **documented** as a restrictive care practice in the hospital's reporting system by **other clinicians where you work/have worked?**	○	

Q138. How frequently have you observed this action being used in the adult mental health inpatient facility where you work/have most recently worked?

Never been observed	Once per year	Once per month	Once per week	Once or more per day	I do not wish to respond to this question
○	○	○	○	○	○

Q139. How frequently have you observed this action being used in the adult mental health inpatient facility that you have experienced?

Never been observed	Once per year	Once per month	Once per week	Once or more per day	I do not wish to respond to this question
○	○	○	○	○	○

Q140.
Case scenario 39: A person is securely locked **alone** in a room.

	Definitely yes	Pr
Do **you think** that this scenario **describes** a restrictive care practice?	○	
Is this action **classified** as a restrictive care practice within the mental health inpatient ward **where you work/have worked**?	○	
Do **you think** that this action should be **recorded** as a restrictive care practice in the hospital's reporting system?	○	
Do you think this action would be **documented** as a restrictive care practice in the hospital's reporting system by **other clinicians where you work/have worked?**	○	

Q141. How frequently have you observed this action being used in the adult mental health inpatient facility where you work/have most recently worked?

Never been observed	Once per year	Once per month	Once per week	Once or more per day	I do not wish to respond to this question
○	○	○	○	○	○

Q142. How frequently have you observed this action being used in the adult mental health inpatient facility that you have experienced?

Never been observed	Once per year	Once per month	Once per week	Once or more per day	I do not wish to respond to this question
○	○	○	○	○	○

Q143.

Case scenario 40: A person is securely locked in a room **while a staff member is present in the room with the person.**

	Definitely yes	Pr
Do **you think** that this scenario **describes** a restrictive care practice?	◯	
Is this action **classified** as a restrictive care practice within the mental health inpatient ward **where you work/have worked**?	◯	
Do **you think** that this action should be **recorded** as a restrictive care practice in the hospital's reporting system?	◯	
Do you think this action would be **documented** as a restrictive care practice in the hospital's reporting system by **other clinicians where you work/have worked?**	◯	

Q144. How frequently have you observed this action being used in the adult mental health inpatient facility where you work/have most recently worked?

Never been observed	Once per year	Once per month	Once per week	Once or more per day	I do not wish to respond to this question
◯	◯	◯	◯	◯	◯

Q145. How frequently have you observed this action being used in the adult mental health inpatient facility that you have experienced?

Never been observed	Once per year	Once per month	Once per week	Once or more per day	I do not wish to respond to this question
○	○	○	○	○	○

Q146.
Case scenario 41: A person is securely locked in a room **together with a group of people.**

	Definitely yes	Pr
Do **you think** that this scenario **describes** a restrictive care practice?	○	
Is this action **classified** as a restrictive care practice within the mental health inpatient ward **where you work/have worked**?	○	
Do **you think** that this action should be **recorded** as a restrictive care practice in the hospital's reporting system?	○	
Do you think this action would be **documented** as a restrictive care practice in the hospital's reporting system by **other clinicians where you work/have worked?**	○	

Q147. How frequently have you observed this action being used in the adult mental health inpatient facility where you work/have most recently worked?

Never been observed	Once per year	Once per month	Once per week	Once or more per day	I do not wish to respond to this question
○	○	○	○	○	○

Q148. How frequently have you observed this action being used in the adult mental health inpatient facility that you have experienced?

Never been observed	Once per year	Once per month	Once per week	Once or more per day	I do not wish to respond to this question
○	○	○	○	○	○

Q149.
Case scenario 42: A nurse applies mechanical restraints to the person **without obtaining consent** from the person or family members / caregivers.

	Definitely yes	Pr
Do **you think** that this scenario **describes** a restrictive care practice?	○	
Is this action **classified** as a restrictive care practice within the mental health inpatient ward **where you work/have worked**?	○	
Do **you think** that this action should be **recorded** as a restrictive care practice in the hospital's reporting system?	○	

	Definitely yes	Pr
Do you think this action would be **documented** as a restrictive care practice in the hospital's reporting system by **other clinicians where you work/have worked?**	○	

Q150. How frequently have you observed this action being used in the adult mental health inpatient facility where you work/have most recently worked?

Never been observed	Once per year	Once per month	Once per week	Once or more per day	I do not wish to respond to this question
○	○	○	○	○	○

Q151. How frequently have you observed this action being used in the adult mental health inpatient facility that you have experienced?

Never been observed	Once per year	Once per month	Once per week	Once or more per day	I do not wish to respond to this question
○	○	○	○	○	○

Q152.

Case scenario 43: A nurse threatens the person with mechanical restraint unless **the person agrees to take the prescribed medication as directed.**

	Definitely yes	Pr
Do **you think** that this scenario **describes** a restrictive care practice?	◯	
Is this action **classified** as a restrictive care practice within the mental health inpatient ward **where you work/have worked**?	◯	
Do **you think** that this action should be **recorded** as a restrictive care practice in the hospital's reporting system?	◯	
Do you think this action would be **documented** as a restrictive care practice in the hospital's reporting system by **other clinicians where you work/have worked?**	◯	

Q153. How frequently have you observed this action being used in the adult mental health inpatient facility where you work/have most recently worked?

Never been observed	Once per year	Once per month	Once per week	Once or more per day	I do not wish to respond to this question
◯	◯	◯	◯	◯	◯

Q154. How frequently have you observed this action being used in the adult mental health inpatient facility that you have experienced?

Never been observed	Once per year	Once per month	Once per week	Once or more per day	I do not wish to respond to this question
○	○	○	○	○	○

Q155.

Case scenario 44: A nurse forcefully confines a person to a seclusion room **based on a report received from the person's family caregivers,** stating that the person intends to physically harm others.

	Definitely yes	Pr
Do **you think** that this scenario **describes** a restrictive care practice?	○	
Is this action **classified** as a restrictive care practice within the mental health inpatient ward **where you work/have worked**?	○	
Do **you think** that this action should be **recorded** as a restrictive care practice in the hospital's reporting system?	○	
Do you think this action would be **documented** as a restrictive care practice in the hospital's reporting system by **other clinicians where you work/have worked?**	○	

Q156. How frequently have you observed this action being used in the adult mental health inpatient facility where you work/have most recently worked?

Never been observed	Once per year	Once per month	Once per week	Once or more per day	I do not wish to respond to this question
○	○	○	○	○	○

Q157. How frequently have you observed this action being used in the adult mental health inpatient facility that you have experienced?

Never been observed	Once per year	Once per month	Once per week	Once or more per day	I do not wish to respond to this question
○	○	○	○	○	○

Reviewing your responses before submission

Q158.

Thank you very much for your participation in this study.

You have now completed the survey. Before proceeding to the next page, you can review or change your responses by clicking the back button.

Once you move to the **next page**, your responses will be automatically submitted online, and you will no longer be able to edit them.
