# Supplementary material for: Discrepancies in classification and reporting of restrictive practices (restraints, seclusion and other coercive measures) in mental health services: multi-scenario analysis of an international survey
Source: BJPsych Open. 2026 May 11;12(3):e131. doi: 10.1192/bjo.2026.11040 (PMC13169055; doi:10.1192/bjo.2026.11040)
Supplement: Belayneh et al. supplementary material 3 — Belayneh et al. supplementary material [file S2056472426110400sup003.docx]

**Supplementary file 3: Frequency of healthcare professionals witnessing the occurrence of potential restrictive care practice scenarios in adult mental health inpatient facilities where they worked**

|  | | How frequently have you observed this action being used in the adult mental health inpatient facility where you work? | | | | | |
| --- | --- | --- | --- | --- | --- | --- | --- |
| # | Scenario, (N) | Never been observed n (%) | Once per year n (%) | Once per month  n (%) | Once per week  n (%) | Once or more per day n (%) | I do not wish to respond to this question |
| 1 | A nurse forcefully confines a person in a locked room to minimize the risk of harm to others, (491) | 105 (21) | 76 (15) | 135 (27) | 103 (21) | 50 (10) | 22 (4) |
| 2 | A nurse forcefully confines a person in a locked room to minimize the risk of self-harm through cutting of body parts, (481) | 165 (34) | 86 (18) | 121 (25) | 62 (13) | 28 (6) | 19 (4) |
| 3 | A nurse forcefully confines a person in a locked room to minimize the risk of suicide, (471) | 170 (36) | 97 (21) | 111 (23) | 60 (13) | 17 (4) | 16 (3) |
| 4 | A person refuses to take prescribed medication so two nurses hold the person onto a bed to facilitate administration of the medication. The medication is safely administered, (465) | 60 (13) | 64 (14) | 121 (26) | 126 (27) | 81 (17) | 13 (3) |
| 5 | A person refuses to take prescribed medication so two nurses hold the person onto a bed to facilitate administration of the medication. The medication is administered but the patient sustains a needle injury during the process, (462) | 233 (50) | 79 (17) | 65 (14) | 44 (10) | 20 (4) | 21 (5) |
| 6 | The person is displaying fearful behaviour by punching the walls of the room. To minimize the risk of self-harm, a security person (guard) firmly holds the person's arm, (458) | 152 (33) | 83 (18) | 93 (20) | 75 (16) | 39 (8) | 16 (4) |
| 7 | The person is displaying fearful behaviour by punching the wall of his room. To minimize the risk of self-harm, a nurse firmly holds the person's arm, (451) | 94 (21) | 98 (22) | 122 (27) | 73 (16) | 41 (9) | 23 (5) |
| 8 | A nurse applies mechanical restraints to a person by keeping the person lying flat on his/her stomach, with his/her face and chest pointing downwards (prone position), (444) | 235 (53) | 66 (15) | 55 (12) | 44 (10) | 21 (5) | 23 (5) |
| 9 | A nurse applies mechanical restraints to a person by keeping the person lying flat on his/her back, with his/her face and chest pointing upwards (supine position), (443) | 120 (27) | 90 (20) | 107 (24) | 69 (16) | 38 (9) | 19 (4) |
| 10 | A nurse locks the individual person's room door to prevent him/her from escaping the hospital, (439) | 180 (41) | 89 (19) | 76 (17) | 41 (9) | 41 (9) | 19 (4) |
| 11 | A nurse locks the whole ward door to prevent a person from escaping the hospital, (437) | 125 (9) | 6 (14) | 61 (14) | 39 (9) | 131 (30) | 20 (5) |
| 12 | A person is kept under mechanical restraint while being transferred between wards to promote safety, (435) | 131 (30) | 97 (23) | 106 (25) | 56 (13) | 28 (6) | 17 (4) |
| 13 | A person is kept under mechanical restraint when staff feel overloaded during busy days to promote safety, (432) | 267 (62) | 43 (10) | 51 (12) | 27 (6) | 17 (4) | 27 (6) |
| 14 | Nurses discuss use of mechanical restraints with the person, but the person refused to give consent to this action. Then, these nurses apply mechanical restraints to the person, (426) | 166 (39) | 77 (18) | 88 (21) | 52 (12) | 26 (6) | 17 (4) |
| 15 | Nurses discuss use of mechanical restraints with the person, but the person refused to give consent to this action. These nurses later engaged in a discussion with the person's family member, and the family member granted consent. The nurses then apply mechanical restraints to the person, (423) | 170 (40) | 81 (19) | 80 (19) | 38 (9) | 35 (8) | 19 (4) |
| 16 | A nurse forcefully confines a person to a seclusion room after witnessing the person attempting to physically harm others, (422) | 81 (19) | 99 (23) | 133 (31) | 74 (18) | 21 (5) | 13 (3) |
| 17 | A nurse forcefully confines a person to a seclusion room after overhearing the person verbally expressing an intention to physically harm others, (422) | 155 (37) | 97 (23) | 89 (21) | 44 (10) | 15 (4) | 22 (5) |
| 18 | The medical team applies mechanical restraints to safely administer injectable medications to a person who exhibits fearful behaviours during hospital admission. The team releases the mechanical restraints immediately after administering the injection, (419) | 129 (31) | 76 (18) | 98 (23) | 60 (14) | 37 (8) | 19 (5) |
| 19 | The medical team applies mechanical restraints to safely administer injectable medications to a person who exhibits fearful behaviours during hospital admission. The team decided to keep the person restrained for one hour after administering the injection, (417) | 162 (39) | 71 (17) | 83 (20) | 53 (13) | 30 (7) | 18 (4) |
| 20 | The medical team threatens the use of mechanical restraint to safely administer injectable medications to a person who exhibits fearful behaviour during hospital admission, (414) | 151 (36) | 62 (15) | 89 (22) | 47 (11) | 41 (10) | 24 (6) |
| 21 | A nurse uses belts to secure a person's arms and legs to the bed as a safety measure to prevent self-harm. This action is taken based on the risk assessment that indicates a higher risk of danger for this person, (410) | 167 (41) | 76 (19) | 83 (20) | 44 (11) | 27 (7) | 13 (3) |
| 22 | A nurse uses belts to secure a person's arms and legs to the bed as a safety measure to prevent self-harm. This action is taken without conducting a risk assessment for this person, (409) | 243 (59) | 59 (14) | 41 (10) | 25 (6) | 22 (5) | 19 (5) |
| 23 | A person is led to a single room to prevent self-harm, and the door is locked, (215) | 95 (44) | 43 (20) | 40 (19) | 20 (9) | 13 (6) | 4 (2) |
| 24 | A person is led to a single room to prevent self-harm, but the door is left unlocked, (214) | 45 (21) | 32 (15) | 60 (28) | 44 (21) | 24 (11) | 9 (4) |
| 25 | The medical team uses devices to restrain a person who is displaying fearful behaviour. The team initially tried sedative medications, but they were not effective, (212) | 69 (33) | 38 (18) | 55 (26) | 31 (15) | 15 (7) | 4 (2) |
| 26 | The medical team uses devices to restrain a person who is displaying fearful behaviour. The team thinks that this is the only option to achieve the desired outcome for that case, but they did not try other approaches first (210) | 110 (52) | 44 (21) | 26 (12) | 15 (7) | 8 (4) | 7 (3) |
| 27 | A nurse applies chain restraints by securing both the person’s wrists and ankles to a bed, (208) | 119 (57) | 18 (9) | 31 (15) | 19 (9) | 11 (5) | 10 (5) |
| 28 | A nurse applies chain restraints by securing one of the person's wrists and ankles to a bed, (208) | 131 (63) | 20 (10) | 25 (12) | 15 (7) | 11 (5) | 6 (3) |
| 29 | A nurse applies chain restraints by securing one of the person’s wrists to a bed, (207) | 137 (66) | 14 (7) | 22 (11) | 17 (8) | 11 (5) | 6 (3) |
| 30 | A nurse securely locks the door of the person's room as a safety measure during the nighttime, (206) | 93 (45) | 32 (16) | 32 (16) | 24 (12) | 20 (10) | 5 (2) |
| 31 | A nurse securely locks the door of the person's room as a safety measure during the daytime, (205) | 95 (6) | 31 (15) | 33 (16) | 19 (9) | 19 (9) | 8 (3) |
| 32 | An individual was kept in a closed, empty room without furniture, windows, light, or toilets to manage agitated behaviour, (205) | 123 (60) | 25 (12) | 29 (14) | 12 (6) | 10 (5) | 6 (3) |
| 33 | An individual is kept in a closed room that is fully furnished with windows, light, and toilets to manage agitated behaviour, (204) | 80 (39) | 30 (14) | 47 (23) | 230 (14) | 12 (6) | 5  (2) |
| 34 | A nurse administers sleep-inducing medication to a person suffering from insomnia. Although the hospital has approved the use of this medication, the nurse increases the amount (dosage) of the medication to achieve an immediate clinical response, (204) | 122 (60) | 25 (2) | 19 (9) | 13 (6) | 11 (5) | 14 (7) |
| 35 | A nurse administers sleep-inducing medication to a person suffering from insomnia**.** This medication is not approved for use in the hospital. However, the nurse decides to administer the medication to achieve an immediate clinical response, (202) | 159 (79) | 11 (5) | 10 (5) | 3 (1) | 8 (4) | 11 (5) |
| 36 | A nurse administers sleep-inducing medication to a person suffering from insomnia. The hospital has approved the use of this medication, and the nurse administers the prescribed dose according to the hospital’s protocol, (201) | 17 (9) | 6 (3) | 22 (11) | 31 (15) | 119 (59) | 6 (3) |
| 37 | A nurse prevents the person from leaving/exiting a designated area or space, (201) | 31 (16) | 30 (15) | 55 (27) | 31 (15) | 47 (23) | 7 (4) |
| 38 | A nurse prevents the person from receiving visits from family, friends, or loved ones, (201) | 80 (40) | 37 (18) | 46 (23) | 20 (10) | 12 (6) | 6 (3) |
| 39 | A person is securely locked alone in a room, (200) | 62 (31) | 34 (17) | 48 (24) | 35 (17) | 17 (8) | 5 (3) |
| 40 | A person is securely locked in a room while a staff member is present in the room with the person, (198) | 99 (50) | 27 (14) | 42 (22) | 13 (6) | 12 (6) | 5 (3) |
| 41 | A person is securely locked in a room together with a group of people, (198) | 128) (4) | 13 (7) | 22 (11) | 12 (6) | 1 (8) | 7 (3) |
| 42 | A nurse applies mechanical restraints to the person without obtaining consent from the person or family members /caregivers, (188) | 71 (36) | 37 (19) | 50 (25) | 21 (11) | 1 (7) | 4 (2) |
| 43 | A nurse threatens the person with mechanical restraint unless the person agrees to take the prescribed medication as directed, (188) | 74 (38) | 36 (18) | 35 (18) | 25 (13) | 18 (9) | 9 (5) |
| 44 | A nurse forcefully confines a person to a seclusion room based on a report received from the person’s family caregivers**,** stating that the person intends to physically harm others, (197) | 107 (54) | 25 (13) | 33 (17) | 15 (8) | 10 (5) | 7 (4) |

**N:** Represents the number of clinicians who completed questions for a specific scenario; **n**= The number of participants who selected specific response options for the outcome questions.

**Additional Set of Case Scenarios:** Items 23-44 were optional questions. Only participants who were interested in answering more questions after completing Items 1-22

**Underlined Statements:** Represent the varying contextual descriptions for parallel comparator scenarios.
