# Supplementary material for: Discrepancies in classification and reporting of restrictive practices (restraints, seclusion and other coercive measures) in mental health services: multi-scenario analysis of an international survey
Source: BJPsych Open. 2026 May 11;12(3):e131. doi: 10.1192/bjo.2026.11040 (PMC13169055; doi:10.1192/bjo.2026.11040)
Supplement: Belayneh et al. supplementary material 4 — Belayneh et al. supplementary material [file S2056472426110400sup004.docx]

**Supplementary file 4: Healthcare professionals ‘personal classification and reporting of potential RCP scenarios compared to actual classification and documentation practices of their in adult mental health inpatient facilities where they work**

|  | 1. Do **you think** that this scenario **describes** an RCP? | | | | | 1. Do **you think** that this action should be **recorded** as an RCP in the hospital’s reporting system? | | | | | 1. Is this action **classified** as an RCP within the mental health inpatient ward **where you work/worked?** | | | | | 1. Would this action be formally **documented** as an RCP in the hospital's reporting system **where you work/worked**? | | | | |
| --- | --- | --- | --- | --- | --- | --- | --- | --- | --- | --- | --- | --- | --- | --- | --- | --- | --- | --- | --- | --- |
| Scenarios | Definitely Yes  n (%) | Probably Yes  n (%) | Probably No  n (%) | Definitely No  n (%) | I do not wish to respond to this question  n (%) | Definitely Yes  n (%) | Probably Yes  n (%) | Probably No  n (%) | Definitely No  n (%) | I do not wish to respond to this question  n (%) | Definitely Yes  n (%) | Probably Yes  n (%) | Probably No  n (%) | Definitely No  n (%) | I do not wish to respond to this question  n (%) | Definitely Yes  n (%) | Probably Yes  n (%) | Probably No  n (%) | Definitely No  n (%) | I do not wish to respond to this question  n (%) |
| #1  N=490 | 309  (63) | 106  (22) | 34  (7) | 38  (8) | 3  (<1) | 305  (62) | 96  (20) | 47  (10) | 37  (8) | 5  (1) | 259  (52) | 107  (22) | 71  (14) | 46  (9) | 8  (2) | 240  (49) | 133  (27) | 70  (14) | 40  (8) | 7  (1) |
| #2  N=480 | 288  (60) | 85  (18) | 43  (9) | 61  (13) | 3  (<1) | 280  (58) | 90 (19) | 45  (9) | 57 (12) | 8  (2) | 237  (49) | 98  (20) | 68  (14) | 68  (14) | 9  (2) | 232 (48) | 115 (24) | 66 (14) | 56 (12) | 11  (2) |
| #3  N=470 | 283  (60) | 86 (18) | 40  (9) | 58  (12) | 3  (<1) | 273 (58) | 88  (19) | 48  (10) | 59  (12) | 2  (<1) | 240  (51) | 95 (20) | 67  (14) | 65 (14) | 3  (<1) | 230  (49) | 109  (23) | 61  (13) | 62 (13) | 8  (2) |
| #4  N=464 | 274 (59) | 97  (21) | 51 (11) | 40  (8) | 2  (<1) | 243  (52) | 112  (24) | 66  (14) | 39  (8) | 4  (<1) | 216 (47) | 103 (22) | 77 (17) | 65 (14) | 3  (<1) | 191 (41) | 114  (25) | 99 (21) | 52 (11) | 8  (2) |
| #5  N=461 | 255  (55) | 91  (20) | 62  (13) | 50  (11) | 3  (<1) | 230  (50) | 103  (22) | 77  (17) | 47  (10) | 4  (<1) | 192  (42) | 99  (21) | 96  (21) | 70  (15) | 4  (<1) | 185  (40) | 106  (23) | 104  (23) | 59  (13) | 7  (1) |
| #6  N=457 | 225  (49) | 121  (26) | 62  (14) | 47  (10) | 2  (<1) | 192  (42) | 128  (28) | 84  (18) | 48  (11) | 5  (1) | 166  (36) | 109  (24) | 106  (23) | 69  (15) | 7  (1) | 147  (32) | 121  (26) | 121  (26) | 61  (13) | 7  (1) |
| #7  N=450 | 204  (45) | 127  (28) | 78  (17) | 39  (9) | 1  (<1) | 176  (39) | 125  (28) | 94  (21) | 50  (11) | 5  (1) | 149  (33) | 118  (26) | 103  25) | 66  15) | 4  <1) | 130  (29) | 125  (28) | 126  (28) | 63  (14) | 6  (1) |
| #8  N=443 | 290  (65) | 69  (16) | 35  (8) | 45  (10) | 4  (<1) | 269  (61) | 76  (17) | 47  (11) | 46  (10) | 5  (1) | 152  (57) | 78  (18) | 54  (12) | 54  (12) | 5  (1) | 239  (54) | 78  (18) | 62  (14) | 56  (13) | 8  (2) |
| #9  N=442 | 288  (65) | 93  (21) | 38  (9) | 20  (4) | 3  (<1) | 267  (60) | 100  (23) | 48  (11) | 24  (5) | 3  (<1) | 257  (58) | 94  (21) | 56  (13) | 31  (7) | 4  (<1) | 243  (55) | 97  (22) | 67  (15) | 31  (7) | 4  (<1) |
| #10  N=438 | 264  (60) | 89  (20) | 44  (10) | 38  (9) | 3  (<1) | 243  (55) | 84  (19) | 64  (15) | 41  (9) | 6  (1) | 207  (47) | 101  (23) | 71  (16) | 52  (12) | 7  (2) | 198  (45) | 106  (24) | 80  (18) | 47  (11) | 7  (2) |
| #11  N=436 | 220  (50) | 94  (22) | 61  (14) | 57  (13) | 4  (<1) | 166  (38) | 110  (25) | 81  (19) | 75  (18) | 4  (<1) | 140  (32) | 94  (22) | 108  (25) | 89  (20) | 5  (1) | 125  (29) | 97  (22) | 109  (25) | 100  (23) | 5  (1) |
| #12  N=434 | 272  (63) | 93  (21) | 35  (8) | 31  (7) | 3  (<1) | 242  (56) | 105  (24) | 53  (12) | 30  (7) | 4  (<1) | 217  (50) | 110  (25) | 59  (14) | 45  (10) | 3  (<1) | 203  (47) | 114  (26) | 62  (14) | 47  (11) | 8  (2) |
| #13 n=431 | 270  (63) | 54  (13) | 39  (9) | 61  (13) | 7  (2) | 245  (57) | 60  (14) | 50  (12) | 67  (16) | 9  (2) | 217  (50) | 71  (16) | 58  (13) | 75  (17) | 10  (2) | 200  (46) | 67  (16) | 75  (17) | 63  (16) | 16  (4) |
| #14  N=425 | 279  (66) | 76  (18) | 37  (9) | 31  (7) | 2  (<1) | 258  (61) | 84  (20) | 42  (10) | 36  (9) | 5  (1) | 240  (57) | 84  (20) | 51  (12) | 45  (11) | 5  (1) | 225  (53) | 94  (22) | 58  (14) | 42  (10) | 6  (1) |
| #15 n=422 | 288  (68) | 82  (19) | 30  (7) | 18  (4) | 4  (<1) | 275  (65) | 85  (20) | 39  (9) | 19  (5) | 4  (<1) | 250  (59) | 89  (21) | 52  (12) | 26  (6) | 5  (1) | 240  (57) | 93  (22) | 51  (12) | 30  (7) | 8  (2) |
| #16  N=421 | 284  (67) | 75  (18) | 40  (10) | 21  (5) | 1  (<1) | 270  (64) | 91  (22) | 42  (10) | 16  (4) | 2  (<1) | 247  (59) | 94  (22) | 52  (12) | 26  (6) | 2  (<1) | 236  (56) | 100  (23) | 53  (13) | 29  (7) | 3  (<1) |
| #17  N=421 | 271  (64) | 70  (16) | 49  (12) | 26  (6) | 5  (2) | 251  (60) | 73  (17) | 55  (13) | 33  (8) | 9  (2) | 222  (53) | 89  (21) | 72  (17) | 32  (7) | 8  (2) | 210  (50) | 96  (23) | 68  (16) | 38  (9) | 9  (2) |
| #18  N=418 | 254  (61) | 78  (19) | 43  (10) | 36  (9) | 7  (2) | 231  (55) | 91  (22) | 46  (11) | 42  (10) | 8  (2) | 208  (50) | 94  (22) | 67  (16) | 41  (10) | 8  (2) | 203  (49) | 89  (21) | 67  (16) | 50  (12) | 9  (2) |
| #19  N=416 | 289  (69) | 74  (18) | 28  (7) | 22  (5) | 3  (<1) | 265  (64) | 82  (20) | 40  (10) | 24  (6) | 5  (1) | 243  (58) | 94  (23) | 47  (11) | 27  (6) | 5  (1) | 231  (56) | 89  (21) | 60  (14) | 30  (7) | 6  (1) |
| #20 n=413 | 167  (40) | 97  (23) | 84  (20) | 58  (14) | 7  (2) | 127  (31) | 90  (22) | 108  (26) | 79  (19) | 9  (2) | 99  (24) | 81  (20) | 134  (32) | 90  (22) | 9  (2) | 95  (23) | 67  (16) | 141  (34) | 101  (24) | 9  (2) |
| #21 n=409 | 291  (69) | 62  (15) | 40  (10) | 20  (5) | 5  (1) | 262  (64) | 75  (18) | 40  (10) | 25  (6) | 7  (1) | 251  (61) | 75  (18) | 50  (12) | 26  (6) | 7  (2) | 232(57) | 86  (21) | 57  (14) | 26  (6) | 8  (1) |
| #22  N=408 | 257  (63) | 44  (11) | 44  (11) | 59  (14) | 4  (<1) | 239  (59) | 56  (14) | 44  (11) | 63  (15) | 6  (1) | 219  (54) | 62  (15) | 54  (13) | 66  (16) | 7  (1) | 207  (51) | 62  (15) | 66  (16) | 65  (16) | 8  (1) |
| #23  N=214 | 143  (67) | 35  (16) | 15  (7) | 21  (9) | 0  (0) | 132  (62) | 43  (20) | 20  (9) | 19  (9) | 0  (0) | 119  (56) | 45  (21) | 27  (13) | 23  (11) | 0  (0) | 117  (55) | 44  (21) | 31  (14) | 21  (10) | 1  (<1) |
| #24  N=213 | 30  (14) | 39  (18) | 83  (39) | 61  (29) | 0  (0) | 28  (13) | 45  (21) | 71  (33) | 69  (32) | 0  (0) | 25  (12) | 37  (17) | 79  (37) | 72  (34) | 0  (0) | 27  (13) | 30  (14) | 83  (39) | 72  (34) | 1  (<1) |
| #25  N=211 | 143  (68) | 43  (20) | 17  (8) | 8  (4) | 0  (0) | 137  (65) | 41  (19) | 23  (11) | 10  (4) | 0  (0) | 134  (64) | 42  (20) | (21  (10) | 14  (6) | 0  (0) | 128  (61) | 42  (20) | 29  (14) | 11  (5) | 1  (<1) |
| #26  N=209 | 140  (67) | 20  (10) | 29  (14) | 20  (10) | 0  (0) | 133  (64) | 25  (12) | 24  (11) | 26  (12) | 1  (<1) | 123  (59) | 31  (15) | 32  (15) | 23  (11) | 0  (0) | 120  (57) | 35  (17) | 30  (14) | 22  (11) | 2  (<1) |
| #27  N=207 | 149  (72) | 27  (13) | 12  (6) | 18  (9) | 1  (<1) | 145  (70) | 22  (12) | 15  (7) | 22  (11) | 1  (<1) | 142  (69) | 23  (11) | 18  (9) | 22  (11) | 2  (<1) | 133  (64) | 32  (15) | 17  (8) | 22  (11) | 3  (1) |
| #28  N=207 | 148  (72) | 33  (16) | 11  (5) | 13  (6) | 2  (<1) | 139  (67) | 39  (19) | 12  (5) | 15  (7) | 2  (<1) | 136  (66) | 37  (18) | 14  (7) | 18  (9) | 2  (<1) | 129  (62) | 46  (22) | 13  (6) | 16  (8) | 3  (1) |
| #29  N=206 | 145  (70) | 34  (17) | 8  (4) | 17  (8) | 2  (<1) | 138  (67) | 34  (17) | 14  (7) | 18  (9) | 2  (<1) | 133  (65) | 32  (16) | 17  (8) | 22  (11) | 2  (<1) | 126  (61) | 40  (19) | 14  (7) | 23  (11) | 3  (1) |
| #30  N=205 | 130  (63) | 37  (18) | 22  (11) | 16  (8) | 0  (0) | 129  63) | 35  (17) | 23  (11) | 19  (9) | 0  (0) | 113  (55) | 36  (18) | 35  (17) | 121  (10) | 0  (0) | 106  (52) | 41  (20) | 35  (17) | 22  (11) | 1  (<1) |
| #31  N=204 | 130  (64) | 37  (18) | 21  (10) | 15  (7) | 1  (<1) | 125  (61) | 36  (18) | 24  (12) | 18  (9) | 1  (<1) | 116  (57) | 34  (17) | 33  (17) | 120  (10) | 1  (<1) | 106  (52) | 37  (18) | 38  (19) | 21  (10) | 2  (<1) |
| #32 n=204 | 139  (68) | 24  (12) | 12  (6) | 26  (13) | 3  (1) | 134  (66) | 23  (11) | 18  (8) | 26  (13) | 3  (1) | 125  (61) | 29  (14) | 19  (9) | 28  14) | 3  1) | 123  (60) | 33  16) | 18  9) | 25  12) | 5  (2) |
| #33  N=203 | 120  (59) | 35  (17) | 35  (17) | 11  (5) | 2  (<1) | 121  (60) | 36  (18) | 31  (15) | 13  (6) | 2  (<1) | 105  (52) | 43  (21) | 37  (18) | 16  (8) | 2  (<1) | 104  (51) | 44  (22) | 39  (19) | 13  (6) | 3  (1) |
| #34  N=203 | 73  (36) | 42  (21) | 37  (18) | 37  (18) | 14  (7) | 75  (37) | 39  (19) | 35  (17) | 39  (19) | 15  (7) | 61  (30) | 34  (17) | 39  (19) | 54  (27) | 15  (7) | 61  (30) | 34  (17) | 45  (22) | 48  (24) | 15  (7) |
| #35 n=201 | 93  (46) | 15  (7) | 32  (16) | 49  (24) | 12  (6) | 85  (42) | 19  (9) | 31  (15) | 52  (26) | 14  (7) | 70  (35) | 21  (10) | 38  (19) | 60  (30) | 12  (6) | 68  (33) | 19  (9) | 44  (22) | 57  (28) | 13  (6) |
| #36 n=200 | 63  (32) | 20  (10) | 34  (17) | 82  (41) | 1  (<1) | 56  (28) | 24  (12) | 33  (17) | 86  (43) | 1  (<1) | 54  (27) | 23  (12) | 32  (16) | 89  (45) | 2  (1) | 54  (27) | 23  (11) | 31  (16) | 90  (45) | 2  (1) |
| #37 n=200 | 90  (45) | 67  (33) | 25  (13) | 16  (8) | 2  (1) | 80  (40) | 60  (30) | 35  (18) | 23  (12) | 2  (1) | 72  (36) | 50  (25) | 55  (28) | 21  (11) | 2  (1) | 67  (34) | 52  (26) | 54  (27) | 24  (12) | 3  (2) |
| #38 n=200 | 95  (48) | 32  (16) | 28  (14) | 41  (21) | 4  (2) | 76  (38) | 34  (17) | 37  (19) | 49  (25) | 4  (2) | 66  (33) | 29  (15) | 51  (26) | 51  (26) | 3  (1) | 56  (28) | 38  (19) | 45  (23) | 56  (28) | 5  (3) |
| #39 n=200 | 128  (64) | 33  (17) | 21  (11) | 17  (9) | 1  (<1) | 127  (64) | 30  (15) | 22  (11) | 21  (10) | 1  (<1) | 119  (60) | 38  (19) | 20  (10) | 21  (11) | 2  (1) | 116  (58) | 38  (19) | 22  (11) | 21  (10) | 3  (2) |
| #40 n=199 | 98  (49) | 39  (20) | 36  (18) | 23  (12) | 3  (2) | 93  (7) | 38  (19) | 340  (20) | 24  (12) | 4  (2) | 81  (41) | 41  (21) | 46  (23) | 28  (14) | 3  (2) | 80  (40) | 40  (20) | 48  (24) | 27  (14) | 4  (2) |
| #41 n=198 | 90  (45) | 38  (19) | 38  (19) | 24  (12) | 8  (4) | 86  (43) | 38  (19) | 36  (18) | 30  (15) | 8  (4) | 75  (38) | 38  (19) | 43  (21) | 34  (17) | 8  (4) | 72  (37) | 38  (19) | 46  (23) | 34  (17) | 8  (4) |
| #42 n=197 | 137  (70) | 26  (13) | 11  (6) | 22  (11) | 1  (<1) | 135  (69) | 24  (12) | 13  (7) | 23  (12) | 2  (1) | 129  (65) | 31  (16) | 12  (6) | 24  (12) | 1  (<1) | 124  (63) | 32  (16) | 11  (6) | 27  (14) | 3  (2) |
| #43 n=197 | 92  (47) | 46  (23) | 33  17) | 25  (17) | 1  (<1) | 75  (38) | 49  (25) | 38  (19) | 33  (17) | 2  (1) | 64  (32) | 44  (22) | 53  (27) | 33  (17) | 3  (2) | 60  (30) | 41  (21) | 53  (27) | 40  (20) | 3  (2) |
| #44 N=197 | 135  (69) | 35  (18) | 13  (7) | 16  (7) | 1  (<1) | 133  (68) | 31  (16) | 20  (10) | 11  (6) | 2  (1) | 120  (61) | 44  (22) | 18  (9) | 13  (7) | 2  (1) | 120 (61) | 40  (20) | 22  (11) | 12  (6) | 3  (2) |

**RCP:** Refers to Restrictive Care Practice, **N**: represents the number of clinicians who completed questions for a specific scenario, **n:** numbers of participants who selected specific response options for the outcome questions
